# VNAR development through antigen immunization of Japanese topeshark (*Hemitriakis japanica*)

**DOI:** 10.3389/fbioe.2023.1265582

**Published:** 2023-09-12

**Authors:** Hiroyuki Takeda, Tatsuhiko Ozawa, Hiroki Zenke, Yoh Ohnuki, Yuri Umeda, Wei Zhou, Honoka Tomoda, Akihiko Takechi, Kimiyoshi Narita, Takaaki Shimizu, Takuya Miyakawa, Yuji Ito, Tatsuya Sawasaki

**Affiliations:** ^1^ Proteo-Science Center, Ehime University, Matsuyama, Japan; ^2^ Department of Immunology, Faculty of Medicine, Academic Assembly, University of Toyama, Toyama, Japan; ^3^ Center for Advanced Antibody Drug Development, University of Toyama, Toyama, Japan; ^4^ Department of Immunology, Graduate School of Medicine and Pharmaceutical Sciences, University of Toyama, Toyama, Japan; ^5^ Fisheries Research Center, Ehime Research Institute of Agriculture, Forestry and Fisheries, Iyo, Japan; ^6^ Graduate School of Biostudies, Kyoto University, Kyoto, Japan; ^7^ Graduate School of Science and Engineering, Kagoshima University, Kagoshima, Japan

**Keywords:** VNAR, Japanese topeshark, phage display, yeast display, biopanning, deep sequencing

## Abstract

The VNAR (Variable New Antigen Receptor) is the smallest single-domain antibody derived from the variable domain of IgNAR of cartilaginous fishes. Despite its biomedical and diagnostic potential, research on VNAR has been limited due to the difficulties in obtaining and maintaining immune animals and the lack of research tools. In this study, we investigated the Japanese topeshark as a promising immune animal for the development of VNAR. This shark is an underutilized fishery resource readily available in East Asia coastal waters and can be safely handled without sharp teeth or venomous stingers. The administration of Venus fluorescent protein to Japanese topesharks markedly increased antigen-specific IgM and IgNAR antibodies in the blood. Both the phage-display library and the yeast-display library were constructed using RNA from immunized shark splenocytes. Each library was enriched by biopanning, and multiple antigen-specific VNARs were acquired. The obtained antibodies had affinities of 1 × 10^−8^ M order and showed high plasticity, retaining their binding activity even after high-temperature or reducing-agent treatment. The dissociation rate of a low-affinity VNAR was significantly improved via dimerization. These results demonstrate the potential utility of the Japanese topeshark for the development of VNAR. Furthermore, we conducted deep sequencing analysis to reveal the quantitative changes in the CDR3-coding sequences, revealing distinct enrichment bias between libraries. VNARs that were primarily enriched in the phage display had CDR3 coding sequences with fewer *E. coli* rare codons, suggesting translation machinery on the selection and enrichment process during biopanning.

## 1 Introduction

Antibodies are used for various purposes ranging from basic research to diagnostics, medicine, and environmental analysis. The development of various technologies for pharmaceutical applications has led to an array of antibodies and antibody-derived molecules, such as bispecific antibodies, antibody-drug conjugates, and chimeric antibody receptors (Liu et al., 2019). Antibody fragments, which have reduced molecular weight while retaining antigen-binding capacity, are frequently employed to modify these antibodies. Antibody fragment formats include Fab, scFv, Fv, VHH, and VNAR (Bates and Power, 2019). VNAR is a single-domain antibody derived from the variable domain of the IgNAR of cartilaginous fishes (Barelle and Porter, 2015). It is the smallest molecule among antibodies and antibody fragments at approximately 12 kDa. Unlike the variable domain of IgG and VHH, which possess the general structure of the Ig domain, VNAR consists of eight β-strands and lacks CDR2 and framework 2. Instead, it has a long, highly diverse CDR3 that facilitates antigen recognition. With their small size, high solubility, plasticity, and thermostability, along with the potential to access epitopes inaccessible to conventional antibodies, VNARs have potential applications in biomedical and diagnostic applications (Griffiths et al., 2013; Kovaleva et al., 2014; Zielonka et al., 2014; Matz and Dooley, 2019; Cheong et al., 2020; Pandey et al., 2022).

As with other antibody fragments, VNARs are obtained by selecting them from VNAR gene libraries using display technology. VNAR libraries include naïve libraries (Liu et al., 2007; Feng et al., 2018; Burciaga-Flores et al., 2023), semi-synthesized (randomized) libraries (Nuttall et al., 2003; Walsh et al., 2011; Häsler et al., 2016; Könning et al., 2016; Könning et al., 2017; Grzeschik et al., 2017; Palacios et al., 2020), synthesized libraries (O’Dwyer et al., 2018; Cabanillas-Bernal et al., 2019), and immunized libraries (Dooley et al., 2003; Bojalil et al., 2013; Camacho-Villegas et al., 2018; Leow et al., 2018; Wei et al., 2021). Due to the induction of antigen-specific IgNAR expression and affinity maturation in sharks inoculated with the target antigen, immunized libraries produced from immunized sharks can be expected to yield a high probability of VNARs with high affinity and specificity (Dooley et al., 2006). However, the difficulty of obtaining and maintaining sharks for immunization and the lack of research tools such as sequence information and antibodies for analysis remains a significant bottleneck in the production of an immunized VNAR library. There are a large number of plate gill species, such as sharks and rays, many of which are endangered due to their small populations. Artificial reproduction is generally difficult for most cartilaginous fishes, and growth requires a considerable amount of time. To consistently produce VNARs using immunized libraries, it is vital to identify cartilaginous fish species that are abundant in nature, readily available in easily manipulated sizes, suitable for long-term rearing and immunization, and capable of inducing antigen-specific IgNAR antibodies through antigen administration.

In this report, we propose the development of VNARs using Japanese topeshark (*Hemitriakis japanica*) (Kamura and Hashimoto, 2004), an unutilized natural resource, as an immune animal. When we started our research, there was no precedent for the immunization of sharks caught in the seas around Japan other than a report of the construction of a semi-synthetic VNAR library of banded houndshark (*Triakis scyllium*) (Kamura and Hashimoto, 2004). We compared several shark species, including closely related species of banded houndshark, and focused on Japanese topeshark for its availability, safety, and competition for food consumption. To demonstrate the potential of Japanese topeshark for VNAR development, we cultivated Japanese topesharks in an artificial environment for extended periods, inoculated them with Venus fluorescent protein as a model antigen, and monitored the induction of antigen-specific antibodies in their plasma.

We also attempted to discuss the potential biases of phage display and yeast display in VNAR development. Several groups have constructed immunized libraries, naïve libraries, and semi-synthetic libraries of VNARs, enriching and selecting antigen-specific VNARs through biopanning. These previous studies have utilized either phage or yeast displays, but no reports have addressed the differences in inherent bias between phage and yeast displays and their impact on the outcomes of VNAR acquisition. We hypothesized that different display technologies, even when generated from the same RNA resource, would result in VNARs with different properties. In this study, we constructed phage display libraries and yeast display libraries using RNA from an immunized Japanese topeshark. We analyzed the diversity of VNAR display libraries using next-generation sequencing in order to comprehensively understand VNAR sequence diversities in the libraries before and after biopanning and to evaluate clonal enrichment during biopanning (Rouet et al., 2018; Barreto et al., 2019). We also prepared recombinant VNARs with major sequences before and after biopanning in the both libraries, observing their antigen binding and stabilities.

## 2 Results

### 2.1 Selection of the immune animal

**FIGURE 1:**
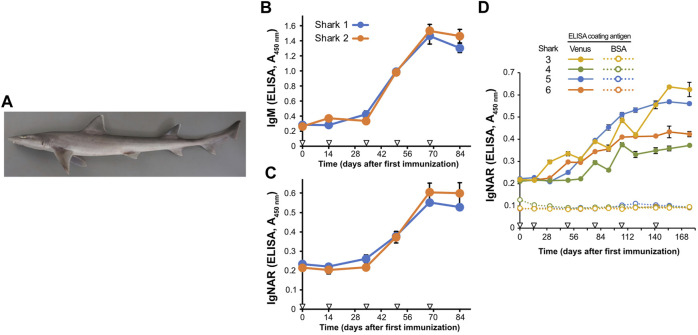
Induction of antigen-specific antibodies in the plasma of Japanese topesharks immunized with Venus protein. **(A)** Japanese topeshark. **(B,C)** IgM and IgNAR antibody titers in immunized Japanese topesharks. Two Japanese topesharks were immunized with Venus once every 2 weeks. Antibody titers in the plasma were quantified via direct ELISA. **(B)** IgM. **(C)** IgNAR. The mean and SD are shown (*n* = 4). Arrowheads indicate immunizations. **(D)** IgNAR antibody titer in long-term immunization. Four Japanese topesharks were immunized with Venus once every 2 weeks. The mean and SD are shown (*n* = 4). Arrowheads indicate immunizations.

We consulted with local fishermen and conducted a preliminary cultivation test on five shark species available in the waters around Matsuyama, Ehime, Japan (Kimura and Hashimoto, 2004): banded houndshark, Japanese topeshark (Figure 1A), starspotted smooth-hound (*Mustelus manazo*), spotless smooth-hound (*Mustelus griseus*), and Japanese bullhead shark (*Heterodontus japonicus*). The first four species, excluding bullhead shark, belong to the family Triakidae (order Carcharhiniformes) and are closely related. Molecular phylogenetic analysis indicates that the banded houndshark and Japanese topeshark are the closest species (López et al., 2006). Bullhead shark, belonging to the family Heterodontidae (order Heterodontiformes), diverges phylogenetically from the other four species.

Following our evaluation, we narrowed our focus to the Japanese topeshark and starspotted smooth-hound. All six of these candidates are docile and present no aggressive behavior towards humans. However, bullhead sharks were ruled out from the candidate list early on due to safety concerns, stemming from their powerful jaws and biting capability. The stable supply of both the banded houndshark and the spotless smooth-hound seemed unattainable due to their low fishery yields in the seas around Matsuyama. As far as we surveyed, Japanese topeshark and starspotted smooth-hound inhabit the coast surrounding Matsuyama and are easily accessible due to their co-capture with other fish in trawl fisheries. They are underutilized fish, rarely consumed as food in Ehime, and infrequently marketed due to their low commercial value. In our preliminary cultivation test, Japanese topesharks exhibited the highest survival rate during prolonged rearing, adapting well to artificial environments and diets. Japanese topeshark and starspotted smooth-hound prey mainly on benthic crawlers using their mortar-shaped teeth; therefore, even when large numbers of individuals were kept in the same pool, they did not injure or cannibalize each other (Kimura and Hashimoto, 2004). Morphologically, the spleen of Japanese topeshark was significantly larger than that of the starspotted smooth-hound (data not shown), which we anticipated would facilitate the yield of IgNAR-expressing plasma cells.

We then conducted immunization studies on both candidate shark species. Recombinant Venus protein was administrated subcutaneously to two Japanese topesharks every 2 weeks to evaluate the induction of the antibody via antigen administration. Plasma samples collected over time from the immunized sharks were assayed for antigen-specific IgM and IgNAR antibodies using Enzyme-Linked Immunosorbent Assay (ELISA) (Figures 1B, C). Both Japanese topesharks demonstrated a significant increase in IgM and IgNAR titers between days 34 and 50, peaked on day 68, and then decreased slightly by day 84. The corresponding rise and fall in antibody titers for both IgM and IgNAR were observed to occur synchronously. Similarly, an increase in antibody titer was observed in starspotted smooth-hounds upon Venus protein administration (Supplementary Figure S1). However, the IgNAR titers in the starspotted smooth-hound were noticeably lower than those in Japanese topesharks, while IgM titers were higher.

In another test, we immunized four Japanese topesharks and monitored the IgNAR titers over an extended period (Figure 1D). Shark 3 exhibited the earliest increase in IgNAR antibody titer at day 31, while Shark 4 showed the slowest response, with an increase only observed at day 78. In all sharks, once antibody titers began to rise, they continued to rise for a significant duration. None of the plasma samples reacted with bovine serum albumin (BSA) in the negative control wells.

### 2.2 Construction of Japanese topeshark VNAR display library

Considering the results mentioned above, we decided to use the immunized Japanese topeshark for the production of VNAR antibodies and then attempted to collect nucleotide sequence information for the VNAR of Japanese topesharks. RNA was extracted from the spleen of several non-immunized Japanese topesharks, and *de novo* RNA sequences were performed. The sequences similar to banded houndshark IgNAR were identified using blast search. Using primers designed from the obtained Japanese topeshark IgNAR fragment sequences, sequences of several VNAR clones were confirmed using 5′-RACE RT-PCR. From the identified VNAR sequences with several variations, several PCR primers were designed for VNAR amplification. We extracted RNA from the splenocytes of immunized shark 1 and used it as a shared material to construct a phage display library and a yeast display library.

**FIGURE 2:**
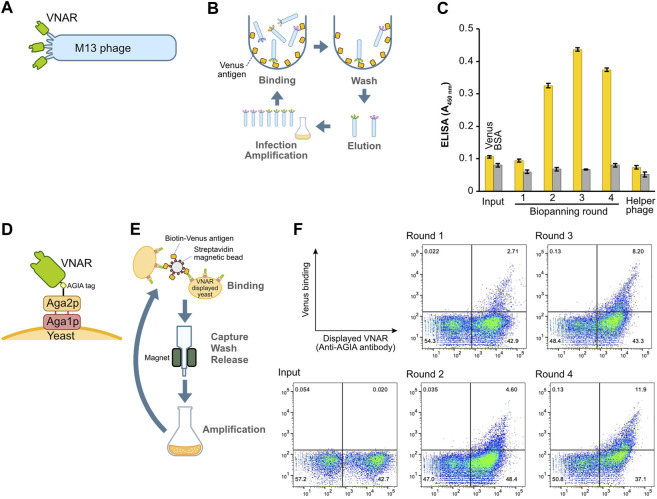
Construction and enrichment of VNAR-displaying libraries from Japanese topeshark. **(A)** Illustration of VNAR-displaying M13 phage. **(B)** The process of biopanning using an immuno tube to enrich for specific VNAR sequences from the phage library. **(C)** Phage ELISA. The wells were coated with Venus or BSA, after which VNAR-, displaying phage at a concentration of 1 × 10^11^ pfu/mL, was applied. **(D)** VNAR displayed on a yeast via Aga1 and Aga2. **(E)** Enrichment of yeast libraries using MACS. **(F)** Flow cytometry. Yeast library was mixed with Venus protein and APC-conjugated anti-AGIA antibody and then applied to a cell analyzer.

In order to construct the phage display library, the VNAR gene was inserted into the pKSTV01 phagemid and fused with the gene 3 protein. This plasmid was then employed to transform *E. coli* strain TG-1, resulting in approximately 2.0 × 10^7^ transformants. The subsequent infection of the transformed TG-1 bacterial cells with a helper phage generated VNAR-displaying M13 phages (Figure 2A). These VNAR-displaying phages were added to an immuno tube in which the Venus protein was immobilized, followed by a period of incubation (Figure 2B). After a thorough washing process, phages bound to the Venus protein were eluted using an acidic solution. The eluted phages were then cultured with *E. coli* to facilitate amplification. This series of steps constituted a single round of biopanning, with a total of four rounds of biopanning performed in this experiment.

To assess the success of the enrichment process for phages displaying antigen-specific VNARs, we performed a phage ELISA (Figure 2C). Phages obtained from libraries prior to the enrichment process (input) and those from the first round of biopanning showed no binding to the Venus-coated wells. However, phages from the second round of biopanning onward exhibited specific binding to Venus, thereby confirming the enrichment of antigen-specific VNAR-displaying phages. Notably, in this set of experiments, there was no non-specific binding observed between the phages and either the BSA or the plate itself.

### 2.3 Construction and biopanning of a yeast display library

The identical cDNA utilized to construct the phage display above was integrated into a yeast display plasmid. Following this, the plasmid was incorporated into the EBY100 yeast strain via electroporation, resulting in the formation of a VNAR-presenting yeast library. In this library, VNAR is fused with Aga2p and presented on the yeast surface through Aga1p (Figure 2D). VNAR-displaying yeast subsequently bound to Venus biotinylated enzymatically and immobilized on streptavidin beads for magnetic beads cell isolation (MACS) (Figure 2E). The magnetic beads, coupled with yeast, were captured using a magnet, and any yeast that remained unbound was washed off. The beads were then retrieved, and the yeast cells bound to these beads were cultured in the medium. This set of procedures constituted a single round of biopanning. In total, this process was performed over four rounds. The results from flow cytometry demonstrated the enrichment of yeast displaying Venus-specific VNARs after the completion of the second round of biopanning (Figure 2F).

### 2.4 Comparisons of VNAR enrichment between phage and yeast libraries

**FIGURE 3:**
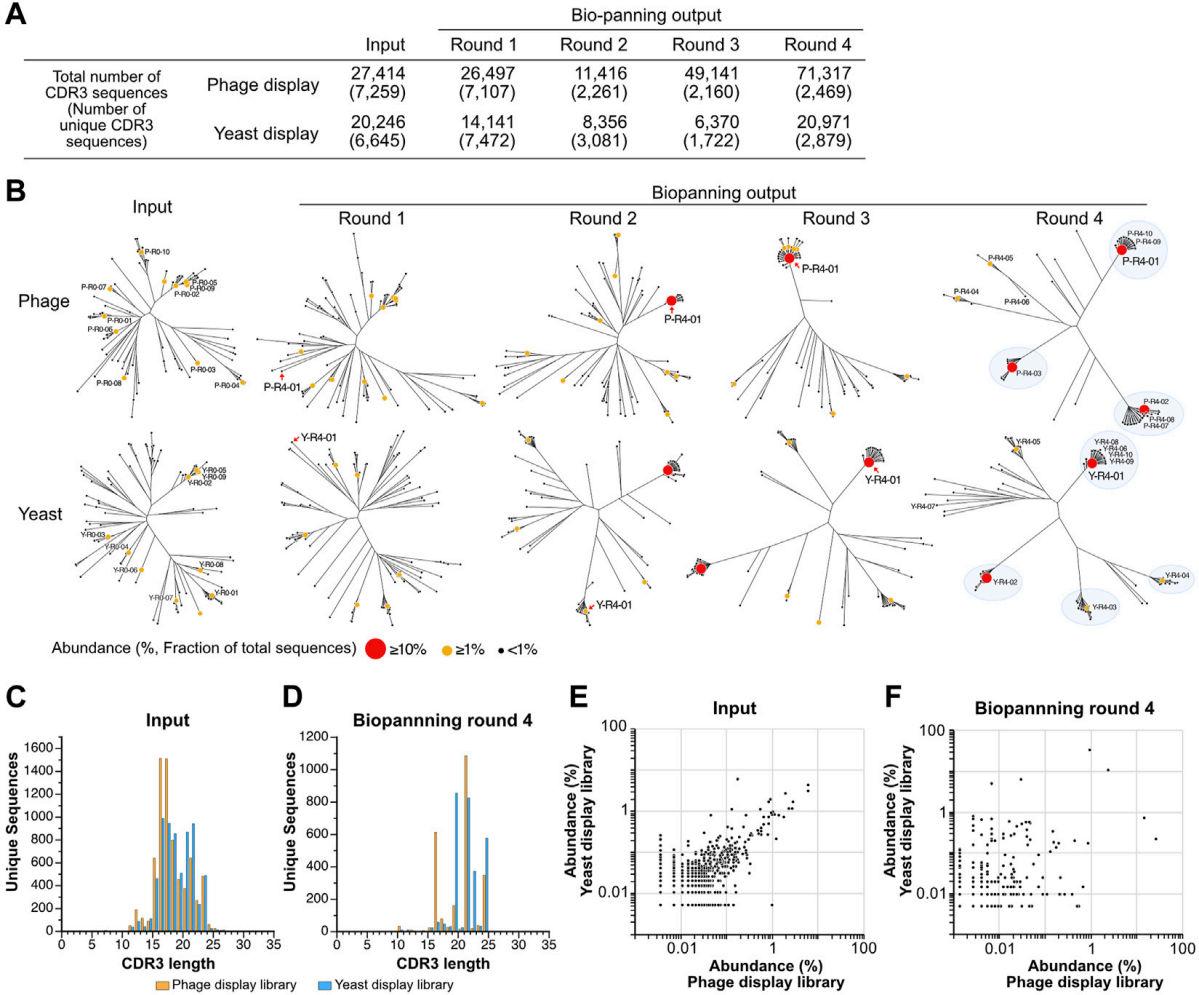
Deep sequencing analysis of VNAR display libraries. **(A)** An overview of the deep sequencing analysis. The total counts of CDR3 reads and unique CDR3 sequences analyzed during each biopanning round are presented. **(B)** The diversity of CDR3 sequences throughout the biopanning process. An amino acid sequence homology analysis was conducted on the top 100 most abundant CDR3 sequences from each biopanning round. Sequence clustering was performed using the Clustal Omega website, and phylogenetic trees were visualized using the FigTree application. Each sequence is represented using a circle, with variations in the size and color of the symbol denoting differences in abundance (the ratio of the number of sequences to the total CDR3 sequences in the round). The top 10 CDR3s in terms of abundance before (input) and after (output, biopanning round 4) biopanning are displayed as follows: Y, yeast; P, phage; R0, the input library; R4, the library enriched via 4-round biopanning; the last number indicates the ranking of abundance. The detailed amino acid sequence and abundance of each CDR3 are available in Supplementary Table S1. Major clusters in round 4 are highlighted with blue circles. **(C,D)** The lengths of CDR3 sequences in both VNAR display libraries. Histograms depict the number of CDR3 sequences for each length. **(C)** Input (phage, 7,259 unique sequences; yeast, 6645); **(D)** round 4 (phage, 2469; yeast, 2879). **(E,F)** A comparison of abundance in both VNAR display libraries for each CDR3 sequence. The abundance of each CDR3 sequence is plotted on the horizontal axis for the phage display and on the vertical axis for the yeast display, respectively. Only CDR3 sequences detected in both libraries are shown, while sequences detected in just one of the libraries are excluded from the analysis. **(E)** Input; **(F)** round 4.

The observation of changes in sequence diversity was achieved through the analysis of VNAR deep sequencing of the libraries, both prior to and following biopanning using next-generation sequencing. Although the deep sequencing analysis identified a significant number of minor variations in regions other than CDR3, such as CDR1, HV regions, and frameworks, we focused our analysis on CDR3 because CDR3 appears to have the highest diversity in VNARs and the greatest contribution to antigen binding (Feng et al., 2018). The sequenced reads containing VNAR sequences were identified, and CDR3 sequences were extracted from them. Among all the sequences analyzed, the longest CDR3 encompassed 31 amino acid residues. The total number of CDR3 sequences in the third round of the yeast library was lowest, with approximately 6.4 × 10^3^ sequences, while the fourth round of the phage library was highest, with 7.1 × 10^4^ sequences (Figure 3A). The number of unique CDR3 sequences detected was roughly equal at 7.3 × 10^3^ and 6.6 × 10^3^ for the input of phage and yeast VNAR display libraries, respectively. A significant decrease in the number of unique CDR3 sequences was noted in the second round of biopanning for both libraries. In the third and fourth rounds, these numbers remained largely unchanged. The number of unique CDR3 sequences in the fourth round was 2.5 × 10^3^ for the phage library and 2.9 × 10^3^ for the yeast library, a difference that is not substantial.

To visualize the changes in sequence diversity throughout the biopanning process, phylogenetic tree analysis was conducted for the top 100 most abundant sequences in the library for each round (Figure 3B; Supplementary Table S1). In this study, we define abundance as the ratio of the read number of a CDR3 to the total number of CDR3 sequences in the library, expressed as a percentage. The phylogenetic trees of the phage library input and round 1 demonstrated relative similarity, with each sequence distributed broadly, thereby avoiding the formation of clusters. A sequence with an abundance exceeding 10% emerged for the first time in round 2 (P-R4-01, 15.3%). Surrounding this highly abundant sequence, several slightly different sequences were noted, forming a small cluster. Round 3 was bifurcated into two groups: a cluster centered on the P-R4-01 sequence and other standalone sequences. In round 4, a major cluster was formed around each of the three sequences (P-R4-01, P-R4-02, and P-R4-03) with an abundance exceeding 10%. Two additional minor clusters centered around P-R4-04 and P-R4-05 were also observed. Each of the top 10 sequences belonged to one of these five clusters. The five sequences that constituted the cluster’s core in round 4 were minor sequences that were not previously included in the top 100 most abundant CDR3 sequences prior to enrichment. The sequence P-R4-01 first appeared at position 56 in round 1 and remained the most abundant sequence from round 2 to round 4.

The enrichment process during the panning of the yeast display library mirrored that of the phage library closely. Three clusters were discernible in round 2, with a fourth cluster appearing in round 3. By round 4, the largest cluster, centered around Y-R4-01, and four smaller clusters, centered around Y-R4-02 through Y-R4-05, were established. Most of the top 10 sequences in round 4 were part of one of these five clusters. However, Y-R4-07 was situated on a branch slightly distinct from these clusters. Compared to round 4 of the phage library, there were more CDR3s that did not belong to major clusters, such as Y-R4-07, in round 4 of the yeast library. Y-R4-01, the most abundant sequence in round 4, was not among the top 100 most abundant CDR3s before enrichment. It ranked 13th in round 1, advanced to 2nd in round 2, and became the most abundant in rounds 3 and 4.

To validate the enrichment trend brought about by biopanning in both libraries, we investigated whether the length of CDR3 varied before and after biopanning. For the phage library input, the median and maximum CDR3 lengths were 17 and 31 residues, respectively, while for the yeast library input, these lengths were 18 and 27 residues (Figure 3C). The shape of both histograms did not conform perfectly to a normal distribution, exhibiting peaks at residues 22 and 24, in addition to the main peak at residue 16. After four rounds of biopanning, the median and maximum lengths were 21 and 24 residues for the phage library and 21 and 26 residues for the yeast library (Figure 3D). The histograms exhibited a comb-like shape, indicating that CDR3s of specific lengths were particularly enriched in both libraries. CDR3s of 21 and 24 residues were prevalent in both phage and yeast. However, those with 16 residues were detected more frequently in the phage library, while those with 19 and 22 residues were more common in the yeast library.

Subsequently, we compared the abundance of individual sequences in both libraries before and after biopanning. The abundance of each CDR3 in the input library was plotted on a graph, with the abundance of each CDR3 in the input library being relatively well dispersed along the diagonal of the XY axis (Figure 3E). The correlation coefficient was 0.710. In contrast, in round 4, each CDR3 was extensively scattered on the plot, and the correlation coefficient was considerably lower at 0.167 (Figure 3F). These results suggest that, although the proportions of each clone in the phage and yeast libraries were initially similar, biopanning led to the enrichment of each CDR3 at different rates.

### 2.5 Different display systems enriched different CDR3 sequences

**FIGURE 4:**
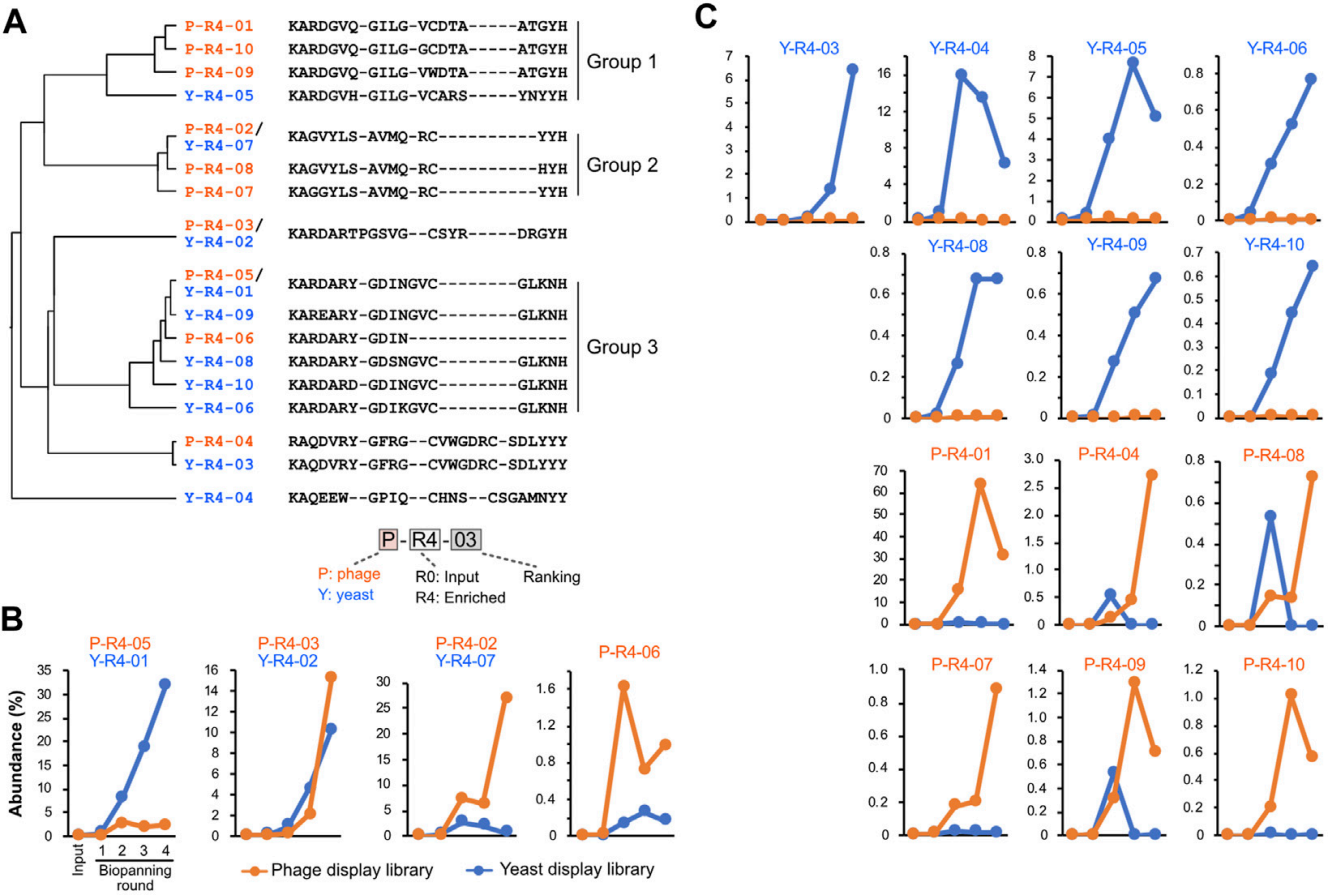
CDR3 sequences enriched by biopanning. **(A)** The sequence and phylogenetic tree of the enriched CDR3 sequences. Sequence alignment was conducted using the MAFFT website, and the phylogenetic tree was visualized using the FigTree application. **(B,C)** Changes in the abundance of CDR3 sequences throughout the biopanning process. Detailed abundance data are available in Supplementary Table S2. **(B)** CDR3s that increased after biopanning in both phage and yeast display libraries; **(C)** CDR3s that increased in either library.

We tracked quantitative changes in representative sequences in both libraries during the biopanning process (Figure 4; Supplementary Table S2). In the input, six of the top ten sequences overlapped between phage and yeast. In the round 4 output of biopanning, three of the top ten sequences were redundant. Homology analysis of the major 17 CDR3 sequences in round 4 broadly divided them into three groups (Figure 4A). Groups 1 and 2 were primarily composed of sequences enriched in the phage library, while group 3 was predominantly populated by sequences enriched in the yeast library.

We graphed the changes in the abundance of each CDR3 sequence throughout the biopanning process. All sequences that were initially abundant in the input libraries saw their abundance significantly reduced by biopanning, regardless of whether they were in the yeast library or phage library (Supplementary Figure S2). Sequences that made the top 10 in the input round of the yeast display all decreased to less than 1% by round 2. In the phage display, all sequences fell below 1% by round 3.

Figure 4B shows the four CDR3s that increased in both the phage library and the yeast library. P-R4-03/Y-R4-02 displayed similar upward curves in both phage and yeast, with abundance increasing from rounds 2 to 3. P-R4-05/Y-R4-01 increased with each round in the yeast library; however, in the phage library, it plateaued in round 2 and did not increase in rounds 3 and 4. Conversely, P-R4-02/Y-R4-07 steadily increased in the phage library, but in the yeast library, it increased in round 2 and then declined slightly in subsequent rounds. The sequence P-R4-06 increased sharply in the phage library in round 2 but decreased in round 3. In the yeast library, it did not make the final top 10, but its presence did slightly increase from rounds 1 to 3.

The 13 sequences illustrated in Figure 4C were enriched exclusively in either phage or yeast. Among these, P-R4-01 was particularly significant, accounting for 63.6% of the total CDR3 in round 3 of the phage library. This proportion dropped to 31% in round 4, likely due to the relative increase in P-R4-02 and P-R4-03. Sequences P-R4-04, P-R4-08, and P-R4-09 were present in high abundance only in round 2 of the yeast library, but declined in rounds 3 and 4.

**FIGURE 5:**
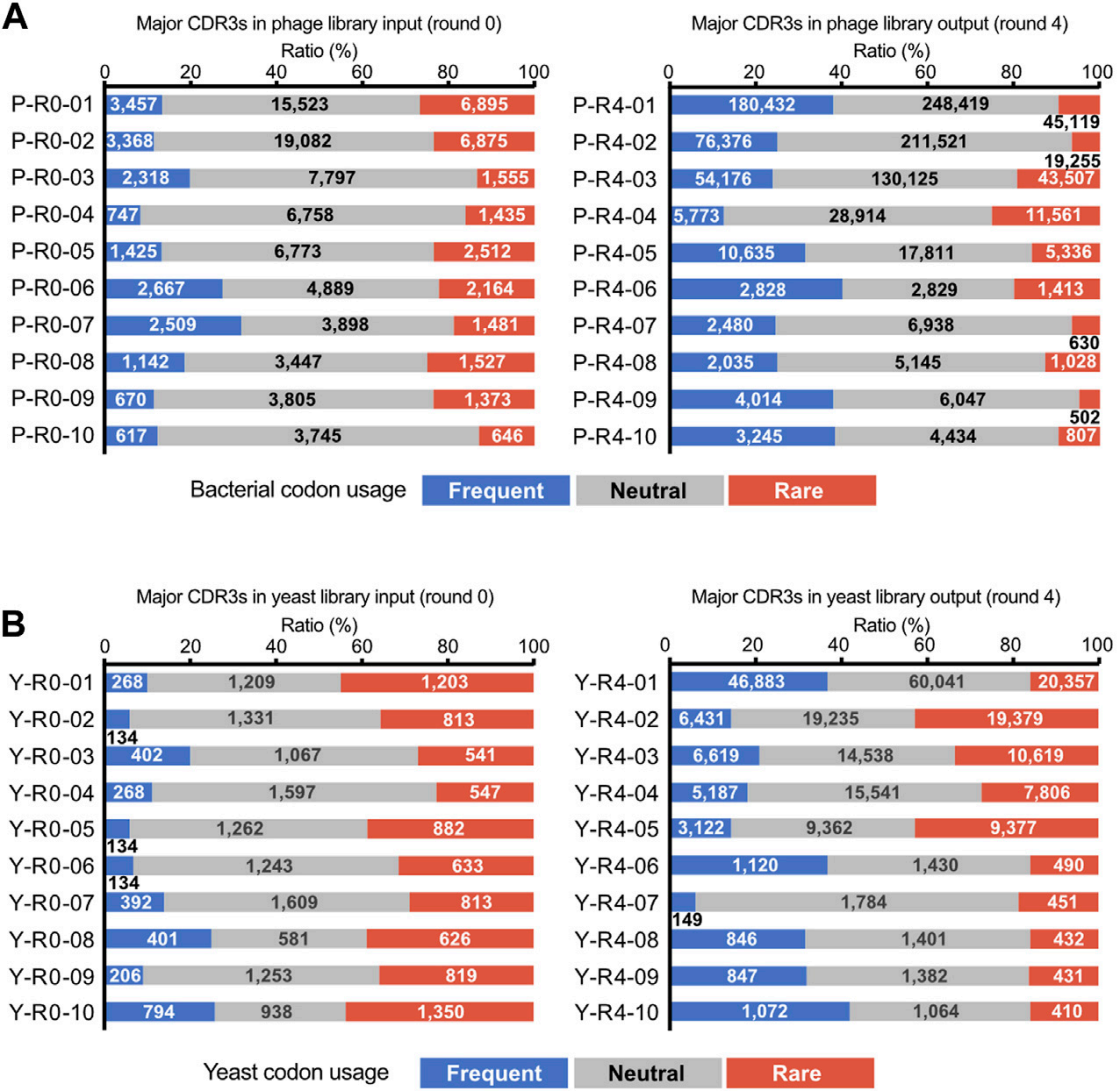
Codon usage analysis. All DNA sequences encoding a CDR3 were extracted from the deep sequencing results. Codon codes that appeared were classified into 3 groups, focusing on the frequency of codon use in either bacteria or yeast^35^. Bacterial codon usage was applied to the analysis of phage library sequences, and yeast codon usage was applied to yeast library sequences. The figures in the bars indicate the number of codons. **(A)** Phage display libraries; **(B)** yeast display libraries.

To assess the impact of differences in the translation systems of the respective presenting libraries on VNAR enrichment results, we paid attention to the codons encoding each CDR3. Many studies have demonstrated that rare codons strongly influence the heterologous expression of proteins (Plotkin and Kudla, 2011). We extracted codon sets from the coding sequences of each CDR3 coding sequences identified using deep sequencing and classified them based on codon usage frequency in either bacteria or yeast (Nakamura et al., 2000). The sequences encoding CDR3, which were major before enrichment, contained an average of 20.5% ± 4.9% *E. coli* rare codons (Figure 5A). After four rounds of enrichment via biopanning, the rare codon content decreased to an average of 12.9% ± 6.8%. CDR3 P-R4-01 and P-R4-02, which were most heavily enriched in phage display library, had only 9.5% and 6.3% of bacterial rare codons, respectively. When the codon usage was observed in yeast, rare codon content decreased from 34.8% ± 7.2% before enrichment to 24.6% ± 11.3% after enrichment. The percentage of rare codons in the CDR3s second (Y-R4-02), third (Y-R4-03), and fifth (Y-R4-05) positions in the output of yeast display library was relatively high, with 43.0, 33.4, and 42.9%, respectively.

### 2.6 The VNARs enriched by biopanning bound to the venus antigen

To verify whether the VNARs enriched through the biopanning process from VNAR-displaying libraries retain their antigen-specific binding ability, we produced recombinant antibodies by fusing VNARs with human Fc and conducted binding assays. Note that only a single clone of VNAR-Fc was prepared and evaluated for each CDR3, which appeared commonly in both the phage and yeast libraries (e.g., P-R4-05 and Y-R4-01). Unfortunately, P-R0-02/Y-R0-02 and P-R4-07 could not be analyzed due to the recombinant VNAR-Fc not being expressed in Expi293F cells.

**FIGURE 6:**
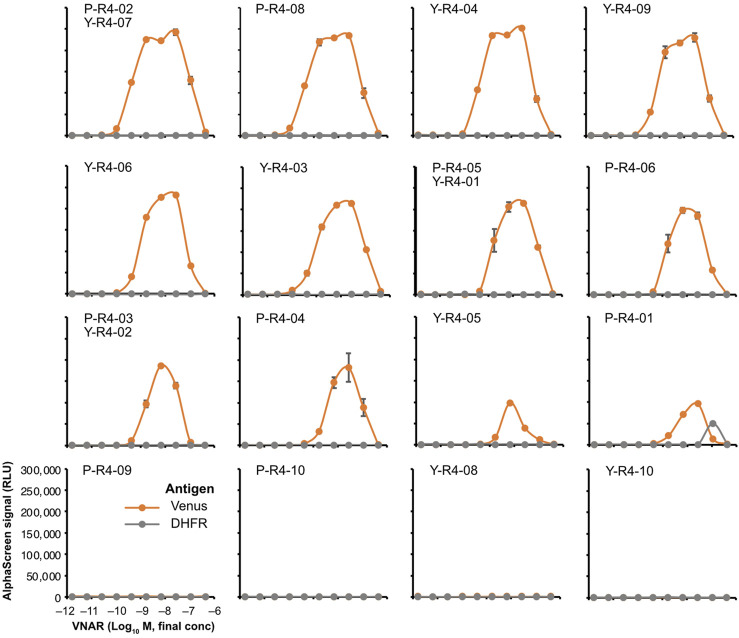
AlphaScreen binding assay of VNARs with enriched CDR3s. VNAR clones with CDR3 sequences enriched using biopanning were, respectively, selected (Figure 3B; Supplementary Table S2) and were fused to human IgG Fc to produce recombinant VNAR-Fc antibodies. The binding of each recombinant VNAR-Fc to biotinylated Venus proteins was detected using AlphaScreen (orange circles and lines). Biotinylated DHFR was used as a negative control (gray circles and lines). The mean and SD are shown (*n* = 4). Graphs were arranged in order of strongest binding response.

The binding assay was performed by using AlphaScreen. None of the VNARs containing CDR3s that were abundant in the input library exhibited any binding to Venus (Supplementary Figure S3). Among the sequences that were highly represented in the biopanning-enriched library, 12 out of the 16 sequences tested showed binding to Venus (Figure 6). The observed decrease in the signal at high concentrations is attributable to the Hook effect. Most VNARs that bound to Venus did not bind to DHFR, a negative control. Only P-R4-01 bound nonspecifically to DHFR at high concentrations. The strongest binding was observed for P-R4-02/Y-R4-07, with an apparent dissociation constant of approximately 0.4 nM. In contrast, the weakest binding was observed for P-R4-01.

### 2.7 Further functional analysis of three VNAR clones

**FIGURE 7:**
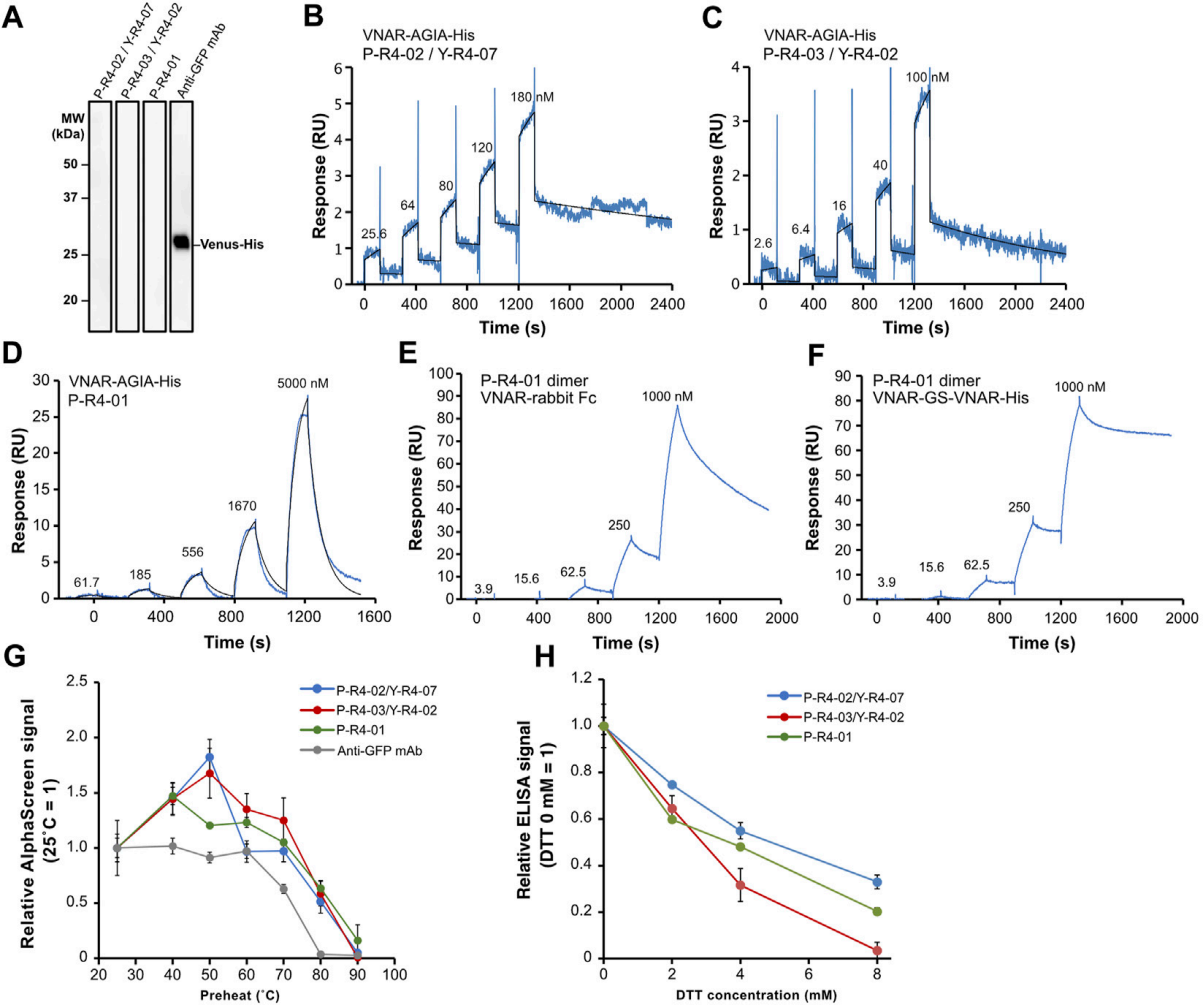
Functional analysis of three recombinant VNAR clones. **(A)** Binding assay to denatured antigen. Membranes on which denatured Venus proteins were blotted were treated with the respective antibodies. **(B–D)** Kinetics assay with surface plasmon resonance. Venus-His protein was immobilized as a ligand on a CM5 sensor chip, and purified VNAR-AGIA-His was applied as an analyte. **(E,F)** Binding assay of bivalent VNAR with CDR3 P-R4-01 to Venus. **(E)** VNAR-rabbit Fc dimer; **(F)** VNAR-GS linker-VNAR-His tandem VNAR dimer. **(G)** Tolerance test for high temperature. VNAR-AGIA-His antibodies were incubated at temperatures ranging from 25°C to 90°C for 1 h. After the samples were allowed to incubate at room temperature for 30 min, binding to Venus was confirmed using AlphaScreen. An anti-GFP mouse monoclonal antibody (clone mFX73, Fuji Film Wako Pure Chemical) was used as a control. The mean and standard deviation are shown (*n* = 4). **(H)** Tolerance test for reducing agents. VNARs were mixed with 2–8 mM DTT and incubated for 30 min. They were then diluted 100-fold in 5% skim milk-PBS and reacted with Venus-coated ELISA plates. The mean and standard deviation are shown (*n* = 4).

Subsequently, we decided to focus on three VNAR clones for further analysis: VNARs containing CDR3 P-R4-02/Y-R4-07, P-R4-03/Y-R4-02, and P-R4-01, respectively. The amino acid sequence homology of these CDR3s is relatively low (Figure 4A). The human Fc-fused VNARs used in the AlphaScreen assay are bivalent, making accurate affinity assessment difficult. In addition, their stability and flexibility are expected to differ from those of monomeric VNARs. To evaluate the binding affinity and plasticity of their VNARs themselves, we prepared VNAR-AGIA tag-His tag and proceeded with functional analysis. As with the Fc fusion constructs, binding of these VNAR-AGIA tag-His antibodies to Venus proteins was confirmed using ELISA. Interestingly, these antibodies did not bind to denatured Venus proteins in Western blotting, indicating that they are structure-recognizing antibodies (Figure 7A).

Kinetic assays were conducted using surface plasmon resonance (SPR), with Venus as the ligand and VNAR-AGIA-His as the analyte. The highest affinity was observed for P-R4-02/Y-R4-07 VNAR, with a dissociation constant (K_D_) of 1.20 × 10^−8^ M (K_a_ = 1.94 × 10^4^ 1/Ms, K_d_ = 2.33 × 10^−4^ 1/s) (Figure 7B). P-R4-03/Y-R4-02 VNAR exhibited nearly the same level of affinity, albeit with slightly faster association and dissociation rates (K_D_ = 1.57 × 10^−8^ M, K_a_ = 5.35 × 10^4^ 1/Ms, K_d_ = 8.42 × 10^−4^ 1/s) (Figure 7C). P-R4-01 VNAR had the weakest affinity among the three, particularly noteworthy for its exceedingly rapid dissociation (K_D_ = 1.32 × 10^−5^ M, K_a_ = 0.95 × 10^3^ 1/Ms, K_d_ = 1.26 × 10^−2^ 1/s) (Figure 7D). When P-R4-01 VNAR was fused to Fc to be a bivalent construct, the association rate barely changed, while the dissociation rate was markedly improved (Figure 7E). Further, when two P-R4-01 VNARs were linked in tandem via a GS linker, the dissociation rate was further decelerated, resulting in a stable binding to the antigen (Figure 7F).

To evaluate the plasticity of each VNAR, they were subjected to high-temperature treatment for 1 h, and subsequently, their binding activities were assessed using AlphaScreen. All three VNARs displayed similar responses. The VNARs treated at temperatures ranging from 40°C to 70°C demonstrated binding signals equal to or greater than those of the samples held at room temperature (Figure 7G). Even after exposure to 80°C, they maintained 37%–66% of their binding activity. Treatment at 90°C for 1 h resulted in these antibodies almost losing their activity. In contrast, when a mouse anti-GFP IgG antibody was treated at 70°C, its binding activity decreased to 63% and almost entirely lost its binding capacity after treatment at 80°C.

Finally, the VNARs were tested for their tolerance to reducing agents (Figure 7H). The VNARs were treated with dithiothreitol (DTT) at concentrations ranging from 2 mM to 8 mM. Due to the potential impact of DTT on the Anti-AGIA antibody used in the AlphaScreen detection of VNARs, an ELISA assay was used to evaluate binding activity of DTT-treated VNARs. Treatment with 2 mM DTT led to a decrease in VNAR activity by 25%–40%. At a concentration of 4 mM DTT, the activity of VNARs was reduced by 45%–68%. P-R4-03/Y-R4-02 VNARs nearly lost all activity after exposure to 8 mM DTT. However, P-R4-02/Y-R4-07 VNAR and P-R4-01 VNAR maintained 33% and 20% of their activity, respectively, after being treated with 8 mM DTT.

## 3 Discussion

In this study, we immunized the Japanese topeshark and starspotted smooth-hound with a protein antigen, subsequently confirming humoral immune responses and antigen-specific IgNAR induction in these sharks. Routine inoculation of Venus protein resulted in an increase in both IgM and IgNAR antibody titers to the antigen (Figure 1; Supplementary Figure S1), suggesting that, similarly to nurse sharks^35^, IgM and IgNAR play roles in humoral immunity in these sharks. Antibody titers increased in both sharks after a minimum of four doses and an 8-week immunization period. A recently published study on immunization of banded houndshark with SARS-CoV-2 RBD showed an increase in IgNAR antibody titers 11 weeks after three immunizations at 3-week intervals (Kim et al., 2023). In contrast, it takes 5–6 months for antibody titers to rise in other shark families, such as nurse sharks, horn sharks, banded wobbegong sharks, and bamboo sharks (Dooley and Flajnik, 2005; Camacho-Villegas et al., 2018; Leow et al., 2018; Wei et al., 2021; Gasperin-Bulbarela et al., 2022). Despite difficulties in making direct comparisons due to variations in antigens and administration conditions, sharks from the family Triakidae appear to be more easily immunized than other shark families. Comparing both species, we finally selected the Japanese topeshark as the immune animal, given its adaptability to the artificial environments, the ease of IgNAR induction, and its large spleen.

To develop VNARs from Japanese topesharks, we had to establish several research resources and tools. We determined the sequences of the IgM heavy chain and the IgNAR genes of the Japanese topeshark through *de novo* RNA sequencing and RACE PCR. Based on the sequence information obtained, we produced polyclonal antibodies specific to the IgM and IgNAR of Japanese topeshark, respectively. By using these antibodies as secondary antibodies in ELISA, we measured the antibody titers in plasma. We also developed a land-based culture method for Japanese topesharks, allowing for their long-term care in an artificial environment. Some sharks were maintained for over a year (data not shown), and we believe they could be sustained for significantly longer. Considering that sharks are generally difficult to breed and require a long time to grow, the Japanese topeshark presents several advantages as an immune animal because they are relatively easy to procure and can be maintained in artificial environments for extended periods. As a result of these technological developments, 10 anti-Venus VNARs were obtained from the immunizing libraries (Figure 6). Functional analysis showed that recombinant VNAR antibodies possess an affinity order of approximately K_D_ = 1 × 10^−8^ M and high plasticity, which allows them to recover their binding activity even when exposed to high temperature or high concentrations of a reducing agent (Figures 7A, B). These results underscore the potential of harnessing Japanese topeshark as a viable source for the practical development of antibodies.

Our biopanning results reveal a marked difference in the enriched CDR3 sequences between phage and yeast display libraries (Figure 3F; Figure 4). This result indicates the existence of a bias in the selection of VNARs due to the different display systems. Both libraries were constructed from an identical RNA sample, and the abundance of individual CDR3 sequences in the library before enrichment (input) exhibited close similarity (Figure 3E). Therefore, we speculated that the bias due to differences in library construction was minimal, and the differences in VNAR selection arose primarily from the biopanning process.

This divergence in VNAR selection could be attributable to the differences in translation machinery between *E. coli* and yeast cells. It is well-known that the frequency of codon usage in open reading frames can affect protein expression levels, with rare codons often reducing translation efficiency (Plotkin and Kudla, 2011). For instance, the CDR3 coding sequences that were predominantly enriched in the phage display (P-04-01, P-04-02) contained fewer rare *E. coli* codons (Figure 5). This result suggests that the codon selectivity of the *E. coli* translation system might have substantially influenced the selection process in the phage display. Conversely, the yeast display library highly enriched even the CDR3 coding sequences containing rare yeast codons (e.g., Y-R4-02, Y-R4-03, Y-R4-05). We speculate that the impact of codon bias in the yeast translation system was relatively minor. Bowley et al. reported that the yeast display was able to isolate many scFvs that the phage display could not (Bowley et al., 2007). Although the study did not directly address differences in the translation machinery of both cell types, it emphasized the significance of “eukaryotic processing of the scFv in the difference in selected clones from yeast and phage display”. Our results lend strong support to this assertion. Strategies for antibody selection should be considered while keeping in mind the bias of the expression system. For example, recent attempts to select antibodies by combining different display systems proposed a beneficial approach for selecting antibodies with high affinity and specificity (Oloketuyi et al., 2021; Teixeira et al., 2021). However, considering our results, the use of display systems with markedly different translation mechanisms in combination may be counterproductive if the objective is to obtain diverse antibodies.

Furthermore, besides differences in translation machinery, the treatment of antigen proteins during the biopanning process might also influence VNAR selection. In the biopanning of the phage display, we directly coated the antigen on the tube surface (Figure 2B). In contrast, in the yeast display, the biotinylated antigen is bound to the magnetic beads via streptavidin (Figure 2E). Rather than directly adsorbing the antigen protein on the solid-phase surface, indirect immobilization via other proteins may facilitate three-dimensional contact between the VNAR and the antigen. Moreover, VNAR-displayed phages are eventually eluted via acid treatment, but if VNAR binding is too strong or VNAR has too high acid tolerance, acid treatment may not elute the phage adequately. In yeast display, yeast cells bound to the beads were directly injected into the medium and cultured without acid elution, offering an advantage to avoid missing useful VNARs.

The VNAR with the P-R4-01 CDR3 sequence was a particularly intriguing subject in this study. This sequence was enriched most efficiently in the phage display library, accounting for over 60% of the output in round 3. As previously discussed, the P-R4-01 sequence appears to be well-suited to the *E. coli* translation machinery. Interestingly, despite its abundance, the affinity of P-R4-01 for Venus is the weakest among the obtained VNARs, with a K_D_ of only 13 µM. Even phage particles are much smaller than yeast cells, and we wondered whether such a weak affinity could sufficiently anchor phages through the dozens of washing steps in the biopanning process. However, a notable improvement in the dissociation rate was observed when the VNAR with CDR3 P-R4-01 was converted into a bivalent form (Figures 7D–F). The M13 phage particle exhibits five copies of the VNAR-fused pIII coat protein, localized at one end of the filamentous phage (Marvin, 1998). It is plausible that two or more P-R4-01 VNAR molecules are in close proximity to each other on the phage surface, creating sufficient binding force to immobilize the phage particle. The results indicate that even phages displaying antibodies with relatively low affinity can compensate for the affinity of the antibody through multivalent interactions and enhance the overall stability of the phage–antigen complex.

In conclusion, we highlight the Japanese topeshark as a promising yet underexplored source for the generation of VNARs. The immunization of Japanese topeshark with protein antigens may allow us to acquire VNARs faster. Although the VNARs of Japanese topeshark have not yet been fully analyzed, comparing their structures and functions with the VNARs of previously studied species such as nurse shark and bamboo shark will reveal whether they possess any advantageous properties for pharmaceutical, diagnostic, or analytical applications. Moreover, we conducted an in-depth analysis of the VNAR selection process from phage and yeast libraries via deep sequencing. We observed that the translation machinery utilized in a display system might affect the selection of VNARs during biopanning. Besides phage and yeast displays, display technologies have been developed using various translation systems and cells. Investigating the binders obtained with these technologies may reveal how translation machinery, secretion systems, and post-translational modifications impact library selection. Such insights could guide the selection of the optimal translation system for diverse antibody display libraries.

## 4 Materials and methods

### 4.1 Antigen preparation

The cDNA encoding the Venus fluorescent protein fused to an N-terminal His-tag was inserted into the pET15b vector using Gibson Assembly (New England Biolabs). *E. coli* BL-21 (DE3) cells, transformed with the expression plasmid, were cultivated in LB medium with 100 μg/mL ampicillin at 37°C until they reached an OD_600_ of approximately 0.55. Induction was carried out with 0.5 mM IPTG, followed by incubation at 37°C for 4 h under shaking conditions. Cells were harvested via centrifugation at 9,100 × g for 10 min at 4°C and subsequently resuspended in lysis buffer containing 50 mM Tris-HCl (pH 8.0), 500 mM NaCl, 5 mM imidazole, ×1 protease inhibitor (Nakalai tesque), 10% glycerol, and 5 mM 2-mercaptoethanol. Cell disruption was conducted via sonication using a sonicator (TOMY, UD-211). Ni Sepharose High Performance resin (Cytiva) was added to the bacterial extract supernatant and mixed using rotation at 4°C for 1 h. The slurry was loaded onto an empty column and washed thrice with lysis buffer. Protein elution was conducted using an elution buffer composed of 50 mM Tris-HCl (pH 8.0), 500 mM NaCl, 10% glycerol, 500 mM imidazole, and 5 mM 2-mercaptoethanol. The purified His-Venus protein was then buffer-exchanged into PBS phosphate-buffered saline using a PD-10 column (Cytiva), portioned into aliquots, and stored at −80°C.

### 4.2 Shark immunization

The animal-handling procedures were approved by the Animal Ethics Committees of Ehime University. Juvenile wild Japanese topesharks, measuring 60–70 cm in length and weighing approximately 0.8 kg, were captured in the coastal region of Matsuyama, Japan and used in this study. Throughout the immunization trials, sharks were housed in a pool filled with natural seawater in the Ehime Fisheries Research Center. Prior to blood collection and immunization procedures, sharks were anesthetized by immersion in seawater containing 2-phenoxyethanol (Fuji Film Wako Pure Chemical). At the conclusion of the experiment, sharks were sacrificed via decapitation under 2-phenoxyethanol anesthesia.

Blood samples and immunizations were conducted at approximately bi-weekly intervals. Incomplete Freund’s adjuvant (Fuji Film Wako Pure Chemical) was mixed and emulsified with the antigen protein using a luer lock syringe and a double hub connector. In each inoculation, an emulsion containing 200 µg of antigen was administered subcutaneously to the fins of each Japanese topeshark. To assess the humoral response of shark IgNAR against the Venus protein, 1 mL of whole blood was drawn from the caudal vein prior to each antigen inoculation. Whole blood was mixed with heparin, and plasma components were separated by centrifugation at 800 × g for 5 min. Plasma samples were stored at −30°C.

### 4.3 Plasma ELISA

To detect shark IgNAR and IgM in ELISA, polyclonal antibody production against banded houndshark IgNAR and IgM was ordered to Kitayama Labes. Peptide antigens were designed based on sequence information of IgNAR and IgM heavy chains in public databases and used for rabbit immunization. The specificity of the polyclonal antibodies was confirmed using Western blotting (Supplementary Figure S4). ELISA was conducted using Maxisorp 96-well immuno plates (NUNC, Thermo Fisher Scientific). The washing procedure was executed using Tris-buffered saline containing 0.5% Tween20 (TBST) and a plate washer (Bio-Tec, AMW-8R). A 50 µL volume of 1 μg/mL Venus or BSA diluted in PBS was added to each well and incubated at 4°C overnight for coating. Subsequent to blocking with 5% skim milk in TBST, shark plasma samples diluted 1/10 with 5% skim milk-TBST were incubated for 1 h at room temperature. After washing, anti-IgNAR or anti-IgM rabbit antiserum diluted 1/50 with 5% skim milk-TBST was added and incubated at room temperature for 1 h. Following further washing, HRP-conjugated anti-rabbit IgG antibody (Cytiva, NA934) diluted 1/5,000 in 5% skim milk-TBST was added and incubated at room temperature for 1 h. The development of color was conducted by adding 1-step Ultra TMB-ELISA substrate (Thermo Fisher Scientific). After terminating the reaction with 1 M HCl, absorbance was measured at 450 nm using a SpectraMax M3 plate reader (Molecular Devices).

### 4.4 RNA extraction

Total RNA was isolated from spleens obtained from sacrificed sharks utilizing the NucleoSpin RNA Plus kit (Takara Bio). Following rDNase (Takara Bio) treatment, RNA was further purified using the NucleoSpin RNA Clean-up kit (Takara Bio). RNA quality was assessed with the Bioanalyzer and RNA 6000 Nano kit (Agilent).

### 4.5 Determination of IgNAR sequence in Japanese topeshark

*De novo* RNA sequence analysis was conducted using total RNA extracted from the spleen of the Japanese topeshark by the Kazusa DNA Research Institute. BLAST analysis was performed with the *de novo* assembled contig sequences as the subjects and IgNAR, IgM, and IgW sequences of Banded houndshark as queries to confirm the partial sequence of the constant region for each antibody. Primers specific for Japanese topeshark IgNAR were designed based on the acquired antibody partial sequences, and 5′and 3′RACE PCRs were executed using the SMARTer RACE 5′/3′Kit (Takara Bio) according to the manufacturer’s instructions.

### 4.6 Construction of phage display library

Reverse transcription was carried out using the extracted total RNA, Oligo (dT)_15_ primer and SuperScript IV Reverse Transcriptase (Thermo Fisher Scientific). A first PCR was conducted using Gene Taq DNA polymerase, and first strand cDNA and primers were designed based on the sequence information obtained from the 5′RACE PCR of Japanese Topeshark IgNAR. A secondary PCR was performed using the first PCR product as a template, adding *Sfi*I and *Spe*I restriction enzyme sites to the 5′and 3′ends of VNAR, respectively. The product was purified via agarose gel electrophoresis following restriction enzyme cleavage. The purified VNAR fragment was ligated to pKSTV01 vector (Rafique et al., 2019) digested with *Sfi*I/*Spe*I (New England Biolabs). The ligation product was purified using diethyl ether, precipitated with ethanol, and dissolved in a small volume of ultrapure water. The resulting phagemids were transformed into TG-1 electrocompetent cells (Lucigen) via electroporation. Transformants were stored at −80°C as glycerol stocks. Library size was determined from the number of transformant colonies on 2TY-AG plates (2TY medium (16 g tryptone, 10 g yeast extract, 5 g NaCl in 1L), 2% glucose, 100 μg/mL ampicillin, and 1.5% agar).

### 4.7 Biopanning of phage-display library

Phage amplification and recovery were conducted as previously reported (Muraoka et al., 2014). Phagemid-transformed *E. coli* (800 µL) in glycerol were lysed and added to 2TY-AG medium (2TY medium, 2% glucose, and 100 μg/mL ampicillin) supplemented with ampicillin and incubated at 37°C until reaching an OD_600_ of 0.6. M13KO7 helper phage (Thermo Fisher Scientific) was added and further cultured overnight in 2TY-AK medium (2TY medium, 100 μg/mL ampicillin, and 50 μg/mL kanamycin). The phage pellet, recovered using centrifugation at 12,000 × g for 30 min at 4°C, was dissolved in a small volume of PBS and clarified with centrifugation and filtration. Diluted phage-infected TG-1 cells were spread on 2TY-AG plates, and phage concentration was determined from the number of colonies observed.

Biopanning was performed using MaxiSorp immuno tubes (Thermo Fisher Scientific). A 2 mL measure of 2.5 μg/mL purified Venus protein was added to a tube and incubated at room temperature for 2 h to coat the surface of the tube bottom. Following blocking with 0.5% BSA, 3 mL of 10^11^ pfu/mL phage was added and incubated at room temperature for 1 h. After washing with PBST, bound phages were eluted with 1.5 mL of 0.1 M glycine-HCl (pH 2.2). Eluted phages were neutralized with 100 µL of 1 M Tris-HCl (pH 9.0) and used to infect log-phase TG-1 cells. Phage-infected TG-1 cells were spread on 2TY-AG plates, and phage concentration was determined from the number of colonies observed.

### 4.8 Phage ELISA

The wells of 96-well titer plates were coated with 50 µL of 1 μg/mL antigen, and after blocking with 0.5% BSA, phages at a concentration of 10^11^ pfu/mL were added and incubated at room temperature for 1 h. Following washing, a mixture of biotinylated anti-M13 phage mouse antibody (Abcam, ab305291) and HRP-conjugated streptavidin (Vector Laboratories, SA-5004-1) was applied. Detection was conducted using 1-step Ultra TMB-ELISA. The color reaction was stopped with 1 M HCl, and absorbance was measured at 450 nm using a Spectra Max M3 plate reader (Molecular Devices).

### 4.9 Construction of yeast display library

The reverse transcription reaction was conducted using SuperScript IV Reverse Transcriptase and oligo (dT)_20_ primer. A first PCR was performed using primeSTAR DNA polymerase (Takara Bio), first strand cDNA, and VNAR-specific primers. The resultant PCR mixtures were diluted 100-fold with water, and the diluted product was applied to the nested PCR. To create pYES3-VNAR, the Aga2 signal sequence, *Nhe*I and *Bam*HI sites, AGIA tag, and Aga2 mature peptide fusion sequence were inserted into the pYES3 vector (Thermo Fisher Scientific). *Nhe*I- and *Bam*HI-digested pYES3-VNAR was mixed with purified and nested PCR products, and the mixture was transformed into electrically competent EBY100 yeast (ATCC) (Chao et al., 2006) using ELEPO21 (Nepa Gene). Four transformations and the dilution plates demonstrated that 1.5 × 10^7^ total transformants were generated for the VNAR library. The yeast was cultured according to previous reports (Chao et al., 2006).

### 4.10 Biopanning of the yeast display library

Libraries were cultured in SDCAA media, passaged, induced in SGCAA media, and subsequently selected as described previously (Chao et al., 2006) using biotinylated Venus coupled to Streptavidin MicroBeads (Miltenyi biotec). Briefly, a VNAR yeast library (2 × 10^9^ cells) was negatively selected with 250 μL Streptavidin MicroBeads for 30 min at 4°C in 5 mL of 0.5% bovine serum albumin and 1 mM EDTA in PBS. Yeast was passed through an LS column (Miltenyi biotec) and washed three times. The flow through was then incubated for 15 min at 4°C with 250 μL Streptavidin MicroBeads pre-incubated with 50 μg/mL biotinylated Venus for 60 min at 4°C. Then, the magnetic beads were passed through an LS column and captured magnetically. After washing the column, the beads were released into SDCAA media, and the yeast cells bound to the beads were cultured. Once yeast reached an OD > 4, they were induced in SGCAA for 2–3 days before an additional selection.

### 4.11 Flow cytometry analysis of yeast

We conjugated allophycocyanin (APC) to antibodies using the Allophycocyanin Labeling Kit-NH_2_ (Dojindo), as described by the manufacturer. Yeast was treated with 150 ng/mL of Venus and 5 μg/mL of APC-conjugated anti-AGIA antibody (Yano et al., 2016) for 20 min at room temperature. The cells were washed twice with PBS containing 0.1% BSA (PBS/BSA), resuspended in PBS/BSA, and analyzed using a FACSCanto flow cytometer (BD Biosciences). Results were analyzed using FlowJo software (BD Biosciences). The gating strategy for flow cytometry is indicated in Supplementary Figure S5.

### 4.12 Deep sequencing of VNAR libraries

Phagemid DNA was extracted from *E. coli* stock infected with the enriched phage library in each round using a Nucleospin Plasmid EasyPure (Takara Bio) and used as a PCR template for deep sequencing. Plasmid DNA was isolated from yeast in each round using the Easy yeast plasmid DNA isolation kit (Takara Bio). The adaptor sequences were appended via the PCR of the purified plasmids using KAPA HiFi HotStart ReadyMix PCR kit (KAPA Biosystems). Deep sequencing was conducted on an Illumina MiSeq sequencer (Illumina) using a MiSeq Reagent Nano Kit v.2 (Illumina). Reads of more than 400 bp were extracted from deep sequencing results and analyzed. Sequences encoding VNAR were selected and their respective CDR3 sequences were extracted. CDR3 alignment was performed using Clustal Omega (Madeira et al., 2022) or MAFFT (Katoh et al., 2017). FigTree (http://tree.bio.ed.ac.uk/software/figtree/) was used to draw phylogenetic trees.

### 4.13 Preparation of recombinant VNAR

VNAR cDNAs prepared by gene synthesis were fused with human Fc or AGIA-His tandem tag and subcloned into the pcDNA3.4 expression vector using Gibson Assembly. VNAR antibodies were expressed using the Expi293F Expression System (Thermo Fisher Scientific, A14635), according to the manufacturer’s instructions. VNAR-Fc antibodies were purified using protein G Sepharose 4 Fast Flow (Cytiva), and then the buffer was exchanged with PBS using PD MiniTrap G-25 (Cytiva). VNAR-AGIA-His antibodies were purified via Ni Sepharose High Performance, and then buffer was exchanged with PBS via PD-10 column. Antibody concentration was determined using the extinction coefficient method (Gasteiger et al., 2005) with a NanoDrop spectrophotometer (Thermo Fisher Scientific). Purified antibodies were frozen and stored at −30°C.

### 4.14 AlphaScreen

The binding assay between VNARs and Venus was conducted via AlphaScreen. Venus and DHFR were fused with the His tag and biotin ligation site (bls) and synthesized using the wheat germ cell-free synthesis system, in which biotin and BirA biotin ligase were added for the biotinylation of bls (Sawasaki et al., 2008). AlphaScreen reactions were conducted in an Optiplate 384 titer plate (PerkinElmer). A 0.5 µL measure of cell-free synthesized and biotinylated protein and VNAR (60 fg to 100 ng) were mixed in a 20 µL dilution buffer (100 mM Tris-HCl, pH8.0, 0.01% Tween20, 100 mM NaCl, and 1 mg/mL BSA). Subsequently, 10 µL of the detection mixture containing 0.1 µL of streptavidin-conjugated AlphaScreen donor beads and 0.1 µL of protein A-conjugated AlphaScreen acceptor beads (PerkinElmer) in the dilution buffer was added to the mixture. After incubation at 26°C for 1 h, the AlphaScreen chemiluminescence signal was detected using an Envision multilabel reader (PerminElmer).

### 4.15 SPR assay

Surface plasmon resonance (SPR) experiment was conducted on a Biacore X100 apparatus (Cytiva). HBS-EP+ (10 mM Hepes-NaOH (pH 7.4), 150 mM NaCl, 0.05% Tween 20, and 3 mM EDTA) was used as a running buffer. The temperature of the flow cells was kept at 25°C during assays. Purified His-Venus was immobilized on the measuring cell of a CM5 sensor chip using amine coupling at 150-200 RU. A single-cycle kinetics assay was performed using purified VNAR-AGIA-His antibodies as the analyte. The sensor chip surface was regenerated by waiting long enough for the analyte to dissociate. The affinity parameter was calculated by using BiaEvaluation software.

### 4.16 Binding assay to denatured antigen

Venus-His recombinant protein was heat-denatured at 95°C for 5 min in SDS-PAGE sample buffer, electrophoresed to 5%–20% SDS-PAGE gel (ATTO), and blotted to PVDF membrane (Cytiva). The blotted membrane was then cut into lanes and treated by anti-Venus VNAR-AGIA-His antibodies, respectively. Following the treatment of anti-AGIA antibody and anti-Rabbit IgG-HRP antibody, the bound antibody was visualized by EzWestLumi plus (ATTO) and ImageQuant LAS4000 imager (Cytiva). An anti-GFP mouse monoclonal antibody (clone mFX73, Fuji Film Wako Pure Chemical) was used as a positive control.

### 4.17 Evaluation of VNAR binding in a reducing environment

VNAR-AGIA-His was mixed with 0–8 mM DTT (Fuji Film Wako Pure Chemical) and 100 μg/mL VNAR-AGIA-His in PBS and then incubated at room temperature for 30 min. Following a 100-fold dilution in 5% skim milk, the antibody–DTT mixture was added to His-Venus coated and 5% skim-milk-blocked immunoplates and incubated. After washing with TBST, VNAR-AGIA-His bound to the antigen was detected using anti-AGIA monoclonal antibody (Yano et al., 2016) and HRP-conjugated anti-rabbit IgG antibody, followed by color development.

## Data Availability

The datasets presented in this study can be found in online repositories. The names of the repository/repositories and accession number(s) can be found below: https://ddbj.nig.ac.jp/search, PRJDB16494.

## Abbreviations

VNAR, variable New Antigen Receptor; ELISA, enzyme-Linked Immunosorbent Assay; BSA, bovine serum albumin; MACS, magnetic beads cell isolation; SPR, surface plasmon resonance; DTT, dithiothreitol; APC, allophycocyanin; BLS, biotin ligation site.
