# Supplementary material for: VNAR development through antigen immunization of Japanese topeshark (Hemitriakis japanica)
Source: Front Bioeng Biotechnol. 2023 Sep 12;11:1265582. doi: 10.3389/fbioe.2023.1265582 (PMC10522858; doi:10.3389/fbioe.2023.1265582)
Supplement: Supplementary file 1 [file DataSheet1.PDF]

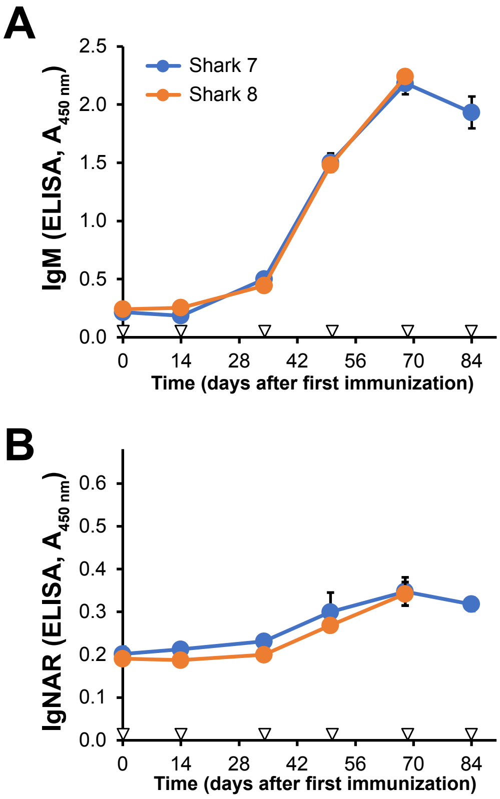

Supplementary Figure S1. IgM and IgNAR antibody titers in immunized starspotted smooth-hounds. Two starspotted smooth-hounds were immunized with Venus protein once every two weeks. The shark 8 died 77 days after immunization. Antibody titers in the plasma were quantified by direct ELISA. (A) IgM; (B) IgNAR. The mean and SD are shown (n=4). Arrowheads indicate immunizations.

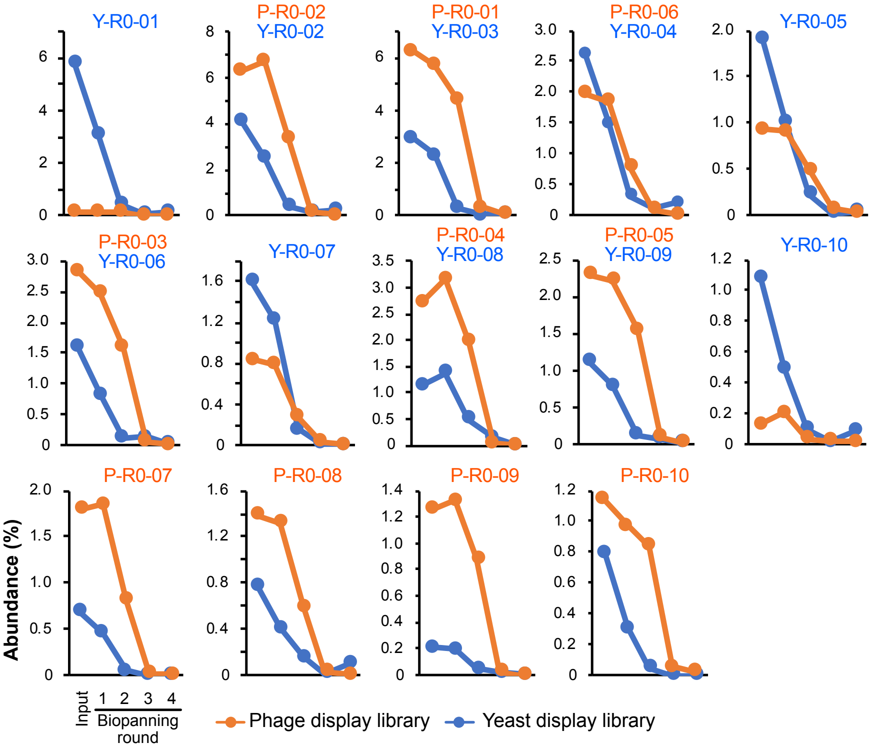

Supplementary Figure S2. Changes in abundance of major CDR3 sequences in input libraries during the biopanning process. The abundance for each biopanning round is shown for the top 10 CDR3s in the input (refer to Fig. 3b). Detailed amino acid sequences and abundance for each CDR3 sequence are available in Supplementary Table S2.

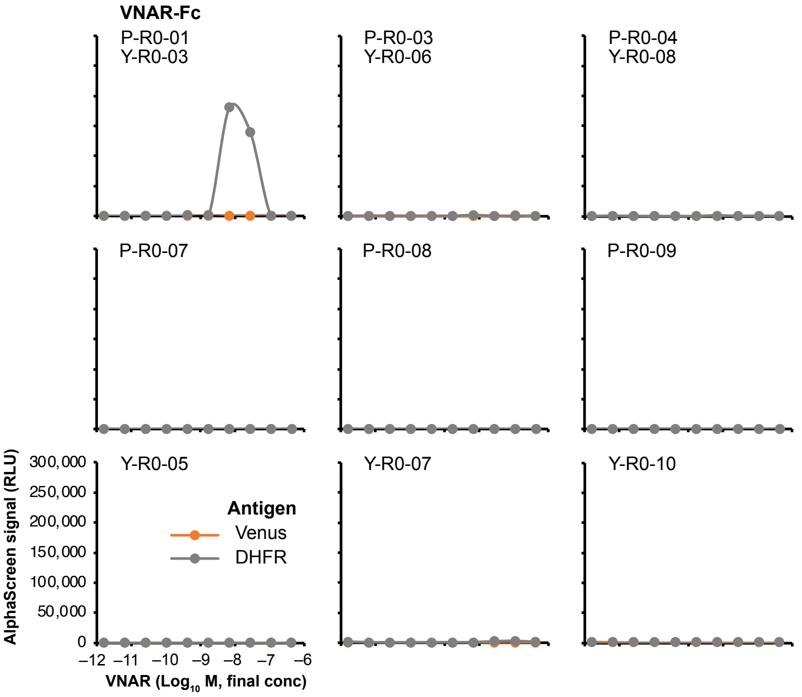

Supplementary Figure S3. AlphaScreen binding assay of VNARs with major CDR sequence in the input libraries. VNAR clones with CDR3s dominant in the input libraries (Fig. 3b and Supplementary Table 2) were respectively selected, and were fused to human IgG Fc to produce recombinant VNAR-Fc proteins. The binding between each recombinant VNAR-Fc and biotinylated Venus proteins was detected by AlphaScreen (orange circle and line). Biotinylated DHFR was used as a negative control (gray circle and line). The mean and SD are shown (n=4).

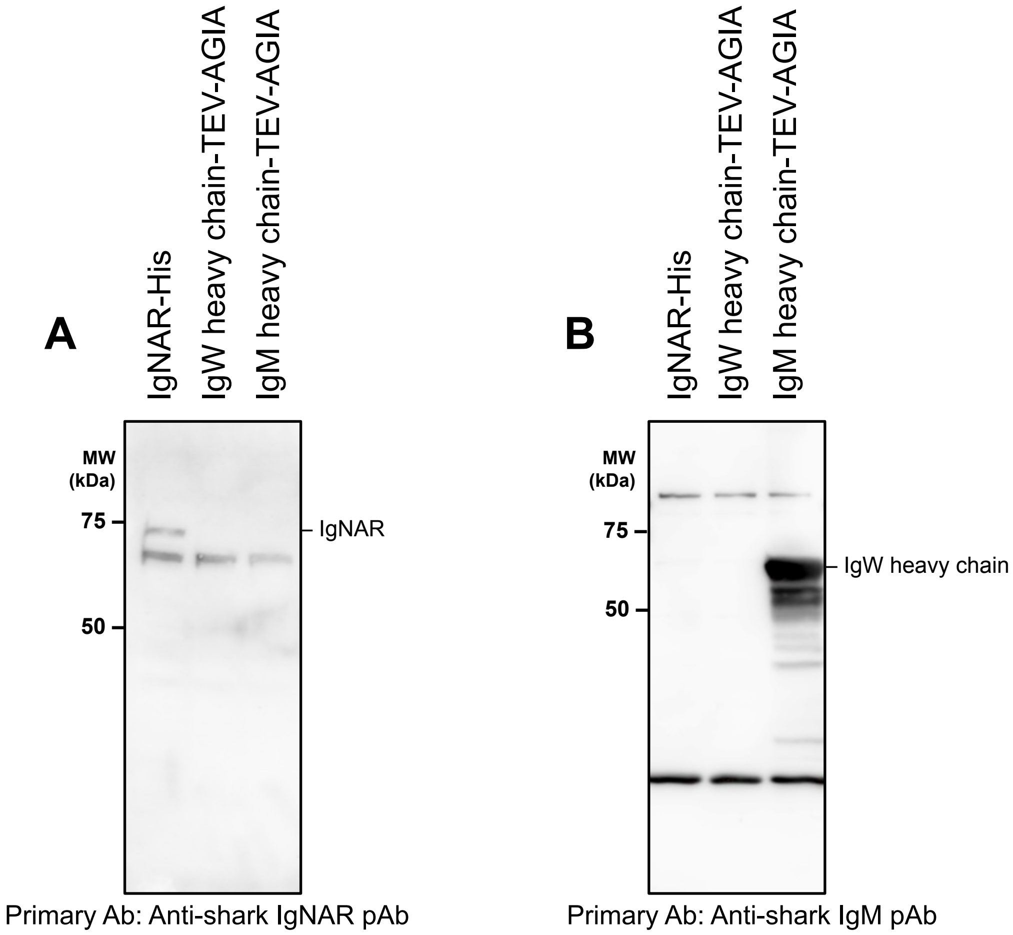

Supplementary Figure S4. Evaluation of rabbit polyclonal antibody against anti-shark antibodies. The banded houndshark IgM heavy chains, IgW heavy chains and IgNAR were synthesized using a wheat cell-free protein synthesis system and electrophoresed as antigens. SDS-PAGE gels (5-20%, ATTO) were used for electrophoresis. Rabbit polyclonal antibody was used as primary antibody and Anti-Rabbit IgG-HRP (Cytiva) as secondary antibody. Visualization was performed by using ImmunoStar LD (Fuji Film Wako Pure Chemical) and ImageQuant LAS4000 imager (Cytiva). (A) Anti-banded houndshark IgNAR polyclonal rabbit antibody. (B) Anti-banded houndshark IgM polyclonal rabbit antibody.

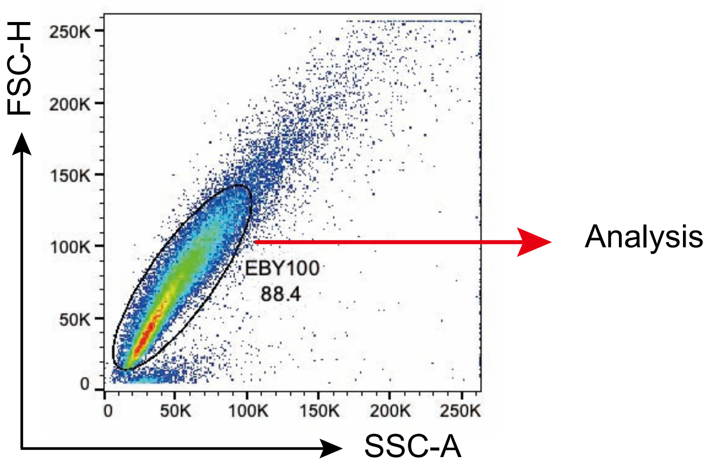

Supplementary Figure S5. Gating strategy for flow cytometry analysis. Gating strategy for Figure 2F. EBY100 gate was set using forward scatter and side scatter. These yeast were used for each experiments.

Supplementary Table S1. Deep sequencing analysis during the enrichment process of the VNAR display library.
For each enrichment process, the top 100 abundant CDR3 sequences were listed.

Phage display library

Ranking	Input Sequence	Input Abundance (%)	Round 1 Sequence	Round 1 Abundance (%)	Round 2 Sequence	Round 2 Abundance (%)	Round 3 Sequence	Round 3 Abundance (%)	Round 4 Sequence	Round 4 Abundance (%)
1	KARDGLRCTTSRYYH	6.29	KARGGTGTSASTRCYYH	6.72	KARDGVQGILGVCDTAATGYH	15.26	KARDGVQGILGVCDTAATGYH	63.65	KARDGVQGILGVCDTAATGYH	31.6
2	KARGGTGTSASTRCYYH	6.29	KARDGLRCTTSRYYH	5.73	KAGVYLSAVMQRCYYH	7.25	KAGVYLSAVMQRCYYH	6.27	KAGVYLSAVMQRCYYH	26.9
3	KARHGGVGSCSSYYY	2.84	KAQKGSCRGNYY	3.16	KARDGLRCTTSRYYH	4.41	KARDARYGDINGVCGLKNH	2.12	KARDARTPGSVGCSYRDRGYH	15.2
4	KAQKGSCRGNYY	2.72	KARHGGVGSCSSYYY	2.48	KARGGTGTSASTRCYYH	3.43	KARDARTPGSVGCSYRDRGYH	2.04	RAQDVRYGFRGCVWGDRCSDLYYY	2.7
5	KARGGTGTSASTRCYYQ	2.30	KARGGTGTSASTRCYYQ	2.22	KARDARYGDINGVCGLKNH	2.91	KARDGVQGILGVCDTAATGYQ	1.56	KARDARYGDINGVCGLKNH	2.5
6	KARDGGGHGVCASSSYYH	1.97	KARDGLKCTQQRSYYH	1.85	KAQKGSCRGNYY	1.97	KARDGVQGILGVWDTAATGYH	1.29	KARDARYGDI	1.0
7	KARDGLKCTQQRSYYH	1.80	KARDGGGHGVCASSSYYH	1.85	KARDARYGDI	1.62	KARDGVQGILGVCDTAATGYH	1.24	KAGGYLSAVMQRCYYH	0.9
8	KARDGRLCVALQQDYH	1.39	KARDGRLCVALQQDYH	1.32	KARHGGVGSCSSYYY	1.59	KARDGVQGILGGCDTAATGYH	1.02	KAGVYLSAVMQRCHYH	0.7
9	KARGGTGTSASTRCYYH	1.25	KARGGTGTSASTRCYYH	1.32	KARGGTGTSASTRCYYQ	1.56	KARDGVQGILGVGDTAATGYH	0.96	KARDGVQGILGVWDTAATGYH	0.7
10	KTRMGTATSCASTYYH	1.14	KARGGTGTSASTRCYYR	1.13	KARMGTATSCSSTYYH	0.89	KARDGVQGILGVCDTAATGDH	0.79	KARDGVQGILGGCDTAATGYH	0.6
11	KARGGTGTSASTRCYYR	1.04	KARGGTGTSVSAGCYYH	1.09	KARGGTGTSASTRCYYH	0.88	KARDARYGDI	0.73	KARDGVQGILGVCDTAATGYQ	0.6
12	KARGGTGTSVSAGCYYH	1.00	KTRMGTATSCASTYYH	0.97	KARGGTGTSASTRCYYR	0.88	KARDGGQGILGVCDTAATGYH	0.52	KAREGYKGDCRYTRYYH	0.5
13	KAQSRVGRVCVGASCSGTYY	1.00	KARDGGGHGVCASSSYYN	0.92	KARDGVQGILGVCDTAATGYQ	0.88	RAQDVRYGFRGCVWGDRCSDLYYY	0.44	KARDARTPGSVGCSYRDGGYH	0.5
14	KARMGTATSCSSTYYH	0.97	KARGGTGTSASTRCYYP	0.91	KTRMGTATSCASTYYH	0.84	KAREGYKGDCRYTRYYH	0.43	KARDGVQGILGVGDTAATGYH	0.5
15	KARDGGGHGVCASSSYYN	0.94	KAQSRVGRVCVGASCSGTYY	0.84	KARDGVQGILGVCDTAATGYH	0.83	KARDGVQGILGVCDTAANGYH	0.39	KARDGVQGILGVCDTAATGYH	0.5
16	KARGGTGTSASTRCYYP	0.93	KAQEQGTAGVCVPRSCSGYYY	0.79	KARDGLKCTQQRSYYH	0.81	KARDGVQGILGVCDTAATCYH	0.38	KAGVDLSAVMQRCYYH	0.4
17	KTREGTHTSCVSTNYH	0.87	KARMGTATSCSSTYYH	0.76	KARDGGGHGVCASSSYYH	0.78	KARDGLRCTTSRYYH	0.33	KAGVYLRAVMQRCYYH	0.4
18	KAQEQGTAGVCVPRSCSGYYY	0.84	KARDGLRCTTSRYYY	0.71	KARDGVQGILGVCDTAATGDH	0.74	KARDGVQGILGVCDTAATGSH	0.33	KARDGVQGILGVCDTAATGDH	0.3
19	KTRMGTATSCAATYYH	0.84	KARDGLRCTTSRYWH	0.66	KARGGTGTSVSAGCYYH	0.71	KARDGVQGILGVCDTAATGFH	0.30	KARDGGQGILGVCDTAATGYH	0.3
20	KARDGLRCTTSRYYY	0.82	KARGGTGTSASTRCYYL	0.62	KAQKGSCRGIYY	0.70	KARDGVQGILGVCDTAATVYH	0.22	KAGVYLSAGMQRCYYH	0.2
21	KARDGLRCTTSRYWH	0.66	KTREGTHTSCVSTNYH	0.59	KAREGYKGDCRYTRYYH	0.65	KARIGTRHDRRCRVPKDRAGYYR	0.21	KAGVYLSAVMQRCYYH	0.2
22	KAREGTDHGDDVCVTQFGAYYH	0.65	KAQEQGTAGVCVPRKCSGYYY	0.58	KARDGRLCVALQQDYH	0.60	KAGGYLSAVMQRCYYH	0.20	KARDARTPGSVGCSYRDRGYQ	0.2
23	KAQKGSCRGIYY	0.59	KARDGKLCVALQQDYH	0.56	KTRMGTATSCAATYYH	0.53	KARDGVQGILGVFDTAATGYH	0.20	KARDARTPGSVGCSYRDRGYH	0.2
24	KAREGTHTSCVSTNYH	0.59	KARDGRVCVRARLLH	0.49	KAQSRVGRVCVGASCSGTYY	0.53	KARDGVQGVLGVCDTAATGYH	0.19	KARDARTPGSGGCSYRDRGYH	0.2
25	KARGGTGTSASTRCYYL	0.59	KAQKGSCRGIYY	0.48	KARDGGGHGVCASSSYYN	0.53	KARDGVKGILGVCDTAATGYH	0.18	KARDGVQGILGVCDTAATCYH	0.2
26	KAQEQGTAGVCVPRKCSGYYY	0.55	KARDGRVCVRLQGGLPH	0.48	KARDGRVCVRLQGGLPH	0.52	KAREGVQGILGVCDTAATGYH	0.18	KARDARTPGRVGCSYRDRGYH	0.2
27	KARDGKLCVALQQDYH	0.51	KTRMGTATSCAATYYH	0.45	KARGGTGTSASTRCYYP	0.48	KARDGVQGILGVCDTAASGYH	0.17	RAQDVRYGFRGCVWGDRGSDLYYY	0.2
28	KARDGRVCVRARLLH	0.49	KARGGTGTSASTRCYYF	0.36	KARDGRVCVRARLLH	0.47	KARDGVQGILGVCDTAATGCH	0.17	KARDARTPGSVGCSYRDRGDH	0.2
29	RARMGTATSCASTYYH	0.36	KTRGGTGTSASTRCYYH	0.34	KARGGTGTSASTRCYYF	0.37	KARGGTGTSASTRCYYH	0.16	KARDGVQGILGVCDTAANGYH	0.1
30	KARDGRVCVRLQGGLPH	0.35	KAREGTDHGDDVCVTQFGAYYH	0.33	KARMGTATSCAASYYH	0.35	KARDGVQGIRGVCDTAATGYH	0.16	KAGVYLSAVMQRCYYQ	0.1
31	KARGGTGTSASTRCYYQ	0.34	KARGGTGTSASTRCYYQ	0.33	KARDGLRCTTSRYWH	0.34	KARDGSRIAVCAPAIGYYH	0.15	KAGVYLSAVMQRCYDH	0.1
32	KARMGTATSCAASYYH	0.33	KARGGTGTSASTRCYYQ	0.33	KARDGLKCTHERSYYH	0.32	KARDGVPGILGVCDTAATGYH	0.14	KAGVYLSAVMQRFYYH	0.1
33	KARGGTGTSASTRCYYF	0.32	KARGTRVCVQL	0.31	KARDGVQGILGVCDTAATGSH	0.32	KARDGVQGILGVCDTADTGYH	0.14	RAQDVRYGFRGCVWGDRCSDLYYY	0.1
34	KAQEHGYSDNVCGLQCVTDYY	0.32	KAREGTHTSCVSTNYH	0.31	KARDGVQGILGVWDTAATGYH	0.32	KARDGVQGILGLCDTAATGYH	0.14	KARDARTPGSVGCSYSDRGYH	0.1
35	KTRGGTGTSASTRCYYH	0.31	KAQEHGYSDNVCGLQCVTDYY	0.29	KAREGTHTSCVSTNYH	0.32	KAGVYLSAVMQRCHYH	0.13	KARDGLRCTTSRYYH	0.1
36	GAQKGSCRGNYY	0.29	KARMGTATSCAASYYH	0.29	KARDGLRCTTSRYYY	0.31	KARDGVQGILGVCETAATGYH	0.13	KARDGVQGILGVCDTAATGSH	0.1
37	KARGGTGTSASTRCYYQ	0.29	KARDGLKCTHERSYYH	0.28	KARDGVQGILGVCDTAATCYH	0.31	KAGVYLRAVMQRCYYH	0.12	KAGVYLSAVMPRCYYH	0.1
38	KARDGLKCTHERSYYH	0.28	KGRGGTGTSASTRCYYH	0.28	KARDGVQGILGVCDTAATGCH	0.31	KARDGVQGIMGVCDTAATGYH	0.12	KAREGVQGILGVCDTAATGYH	0.1
39	KARDGGSLGSGRVCSTAGYYH	0.27	GAQKGSCRGNYY	0.27	KAREGITACDSGGYYH	0.29	KARDGVQGILGVCYTAATGYH	0.11	KAGVYLSALMQRCYYH	0.1
40	KARDGLRCTTARYWH	0.27	KARGGTGTSASTRCYYP	0.27	KTREGTHTSCVSTNYH	0.29	KARDGVQGILGVCDKAATGYH	0.11	KARDGVQGILGVCDTAATGFH	0.1
41	KAREGTVVDVCVEGYYH	0.26	RARMGTATSCASTYYH	0.26	KAQEQGTAGVCVPRSCSGYYY	0.28	KARGGTGTSASTRCYYQ	0.11	RAQDVRYGFRGWVWGDRCSDLYYY	0.1
42	KARGGTGTSASTRCYYP	0.26	KAQEPVYLSGIGCGADRCSGHYY	0.24	KARDGVQGILGVCDTAATGFH	0.27	KAGVDLSAVMQRCYYH	0.10	KARDGVQGILGVCDTAATGCH	0.1
43	KARGTRVCVQL	0.24	KAREGTVVDVCVEGYYH	0.24	KAQEQGTAGVCVPRKCSGYYY	0.25	KARDARTPGSVGCSYSDRGYH	0.10	KARDGVQGILGVFDTAATGYH	0.1
44	KARGGTGTSASTRCYFQ	0.23	KARDGLKCTRERSYYH	0.23	KARDGNVCVKLRSYYH	0.25	KARDGVQGILGVCDPAATGYH	0.09	KAGVYLSAVMQRGYYH	0.1
45	KAQEPVYLSGIGCGADRCSGHYY	0.20	KAQEHRTCPRGGSCSGPYY	0.21	KARDGKLCVALQQDYH	0.25	KARDGVQGILGVCDSAATGYH	0.09	KARDARTPGGVGCSYRDRGYH	0.1
46	KARDADGEGVCAERYYH	0.20	KAQEPVYLSGIGCGADSCSGHYY	0.20	KARDGVQGILGVCDTAATVYH	0.25	KARDGVQGILGVCDAAATGYH	0.08	KARDGSRIAVCAPAIGYYH	0.1
47	KGRGGTGTSASTRCYYH	0.20	KARDGLRCTTARYWH	0.20	KCRGGTGTSASTRCYYH	0.25	KARDGGGHGVCASSSYYH	0.08	KARDGVKGILGVCDTAATGYH	0.1
48	KARGGTGTSASTRGYYH	0.19	KARDARYGDINGVCGLKNH	0.20	KARGGTGTSASTRCYYL	0.24	KARDARTPGSVGCSYRDGGYH	0.07	KARDGVQGIMGVCDTAATGYH	0.1
49	KARDGGGHGVCASSSYYQ	0.18	KARDGRLCSTHQGYYH	0.20	KARDGPCSGIYYH	0.23	KARDGVQGILGVCDTATTGYH	0.07	RAQDVRYGFRGGVWGDRCSDLYYY	0.1
50	KAQSRVGRVCVGASCSGPYY	0.18	KARDGGGHGVCASSSYYQ	0.19	KARDARTPGSVGCSYRDRGYH	0.22	KARDGVQGSLGVCDTAATGYH	0.07	KAGVYLSAVMQLCYYH	0.1
51	KARDGLKCTRERSYYH	0.18	KARDGGSLGSGRVCSTAGYYH	0.19	KARGTRVCVQL	0.22	KARGGTGTSVSAGCYYH	0.07	KAGVYLSAVMQSCYYH	0.1
52	KTREGGRGSCVSGHYH	0.18	KARDCRSGLYYH	0.18	KARDARTPGSVGCSYSDRGYH	0.21	KARDGVQGILGVCDTASTGYH	0.07	KARDGVPGILGVCDTAATGYH	0.1
53	KARGGTGTSASTRCYYN	0.17	KARGGTGTSASTRGYYH	0.18	KARDGLKCTQKRSYYH	0.21	KARDGVQGILGVCDTSATGYH	0.07	KAGLYLSAVMQRCYYH	0.1
54	KARDGGGNVCQHGDYH	0.17	KARDADGEGVCAERYYH	0.18	KARDGVQGILGGCDTAATGYH	0.21	KARGGTGTSASTRCYYP	0.07	KARDGVQGILGVCDTAASGYH	0.1
55	KARDGLRCTTSRYNH	0.17	KARDDDLCVRLHVGGYYH	0.18	KARDARYGDINGVCGLKTH	0.20	KARYGVQGILGVCDTAATGYH	0.07	KARDGVQGILGVCDTADTGYH	0.1
56	KARDGLRGTTSRYYH	0.17	KARDGVQGILGVCDTAATGYH	0.18	KARDARYGDINGVCGLKNH	0.19	KTRMGTATSCAATYYH	0.07	RAQDVRYGFRGCGWGDRCSDLYYY	0.1
57	KARDGRLCSTHQGYYH	0.17	KAQSRVGRVCVGASCSGPYY	0.18	KARDARYGDINGVCGLKNQ	0.19	KARAGVQGILGVCDTAATGYH	0.07	KARDGVQGIRGVCDTAATGYH	0.1
58	KTRMGTATSCAASYYH	0.17	KTREGGRGSCVSGHYH	0.18	KARDGRSCTGLRGHYH	0.19	KARDARYGDINGVCGLKDH	0.07	KARDGVQGVLGVCDTAATGYH	0.1
59	KARDGNVCVKLRSYYH	0.16	KAKKGSCSGNYY	0.17	KARDGVQGILGVCDTAANGYH	0.19	KAGVYLSAVMQRCYYH	0.06	KARDGVQGILGVCDPAATGYH	0.1
60	KAKKGSCSGNYY	0.16	KAQKGSCSGNYY	0.17	KARGGTGTSASTRCYYQ	0.19	KARDGLQGILGVCDTAATGYH	0.06	KAGVYLSAVMKRCYYH	0.1
61	KARGGTGTSASTTCYYH	0.16	KARDGLRCTTSRYNH	0.17	KAQKGSCSGIDY	0.18	KARDGVQGILGVCATAATGYH	0.06	KARDGVQGILGVCDKAATGYH	0.1
62	KAHMGTATSCAATYYH	0.16	KARDGRLCSTRNYYH	0.17	KARDGVQGILGVGDTAATGYH	0.18	KARDGVQGILGVCDTEATGYH	0.06	KAGVYRSAVMQRCYYH	0.1
63	KAQEHRTCPRGGSCSGPYY	0.16	KARGGTGTSASTRCYFQ	0.17	KAREGTDHGDDVCVTQFGAYYH	0.18	KARHGGVGSCSSYYY	0.06	KARDARYGDINGVCGLKNQ	0.1
64	KARDAVHGDCASGSVYYH	0.16	KARDGGGHGVCASSSYYH	0.16	KARGGTGTSASTRCYYQ	0.18	KARMGTATSCSSTYYH	0.06	KAGVYLNAVMQRCYYH	0.0
65	KARDCRSGLYYH	0.15	KARDGLRCTTSRYDH	0.15	KTREGGRGSCVSGHYH	0.18	KARDGVQGILGVCDTAATGYP	0.06	KAGVYLSAVMQGCYYH	0.0
66	KARGGTETSASTRCYYH	0.15	KAQKGSCKGNYY	0.15	KAAKGSCRGNYY	0.18	KARDARYGDINGVCGLKNQ	0.05	KARDARYGDINGVCGLKDH	0.0
67	KARDACNRVQQLQDYH	0.15	KVRMGTTTSCASTYYP	0.15	KAGGYLSAVMQRCYYH	0.18	KAQKGSCRGIYY	0.05	KARDGVQGILGVCDTAATVYH	0.0
68	KARDGLKCTQKRSYYH	0.15	KARGGTETSASTRCYYH	0.15	KARDGVQGILGVCDTAASGYH	0.17	KARDARYGDINGVCGLKNH	0.05	KARDGVQGILRVCARATGGYYH	0.0
69	KARHGGVNSCSSYYY	0.15	KARGGTGTSASTTCYYH	0.15	KAQSRVGRVCVGASCSGPYY	0.16	KARDGVQGILGVCDTAATDYH	0.05	KAGVYLSAVIQRCYYH	0.0
70	KGRGGTGTSVSAGCYYH	0.15	KARDGGGHGVCASSSYYK	0.14	KVRMGTTTSCASTYYP	0.16	KARDGVQGILGVCDTAATGDQ	0.05	KARDGVQGILGVCYTAATGYH	0.0
71	KAQKGSCSGNYY	0.14	KARDGRLCSAATVRKHH	0.14	RARMGTATSCASTYYH	0.16	KARDGVQGILGVCDTAATGYQ	0.05	RAQDVRYGFRGCVWGDRCRDLYYY	0.0
72	KARGGTGTSASSRCYYQ	0.14	KARDGRLCVSVQGDYH	0.14	KAGVYLSAVMQRCHYH	0.15	KTRMGTATSCASTYYH	0.05	KAGVYLSAVRQRCYYH	0.0
73	KARDGGGHGVCASSSYYK	0.13	KARGGTGTSASTRCYYN	0.14	KARGGTGTSASTRCYYR	0.15	KARDGVQGILRVCARATGGYYH	0.05	KARDARPPGSVGCSYRDRGYH	0.0
74	KTHMGTATSCASTYYH	0.13	KCRGGTGTSASTRCYYH	0.14	KARMGTGTSCSSTYYH	0.15	KARDGVQGILGVCDTAATGHH	0.05	KAGVNLSAVMQRCYYH	0.0
75	KAQEPVYLSGIGCGADSCSGHYY	0.13	KVRGGTGTSASTRCYYH	0.14	KTRMGTATSCAASYYH	0.14	KARDGVQGILGVCDTAATGYH	0.14	KARDARTPGSVGCSYRDRGFH	0.0
76	KARDGGGHGVCASSSYYH	0.13	KARDGLRGTTSRYYH	0.14	KAGVDLSAVMQRCYYH	0.14	KAQKGSCRGNYY	0.13	KARDGVQGILGVCDSAATGYH	0.0
77	KARMGTATSCAATYYH	0.13	KARDGNVCVKLRSYYH	0.14	KAQEHRGRMCVASCSGNY	0.14	KARDGVQGFLGVCDTAATGYH	0.13	KARDGVQGILGVCETAATGYH	0.0
78	KARMGTATSCATTYYH	0.13	KARGGTGTRASTRCYYH	0.14	KAQEHRTCPRGGSCSGPYY	0.13	KARDGVQGILGVCDTAAKGYH	0.13	RAQDVRYGVRGCVWGDRCSDLYYY	0.0
79	KCRGGTGTSASTRCYYH	0.13	KAGVYLSAVMQRCYYH	0.13	KARGGTETSASTRCYYH	0.13	KAGVYLSAVMQRFYYH	0.13	KAGVFLSAVMQRCYYH	0.0
80	RTRMGTATSCASTYYH	0.13	KARDAVHGDCASGSVYYH	0.13	RAQDVRYGFRGCVWGDRCSDLYYY	0.13	KARDGLRCTTSRYYY	0.13	KARDARTPGSVGCSYRERGYH	0.0
81	KARMGTATSCASTYYH	0.12	KARGQRGICRYYH	0.13	KARDGLRCTASRYHH	0.13	KARDGVQGILGACDTAATGYH	0.12	KARDARTPGSVGCSYRDRGSH	0.0
82	RAREGTHTSCVSTKYH	0.12	RAREGTHTSCVSTKYH	0.12	KARDGRLCVALQQDVH	0.13	KARDGVQGILGVWDTAATGDH	0.12	KARDGVQGILGLCDTAATGYH	0.0
83	KARDGLRCTTSRYDH	0.12	KARDAPRGSGSLQLQCGYH	0.13	KAREGTVVDVCVEGYYH	0.13	KARDGVQGILGVWDTAATGYQ	0.12	KARGGTGTSASTRCYYH	0.0
84	KARDGRLCVSVQGDYH	0.12	KARDGRLCSTRNYYH	0.12	KARPGGVGSCSSYYY	0.13	KARGGTGTSASTRCYYR	0.12	KARDARYGDINGVCGLKNH	0.0
85	KARDGLKCTQRRSYYH	0.12	KARGGTGTSASTRCYYR	0.13	GAQKGSCRGNYY	0.13	KARDGLKCTHERSYYH	0.11	KARDARTPGSVGCSSRDRGYH	0.0
86	KAQKGSCKGNYY	0.11	KGRGGTGTSVSAGCYYH	0.13	KARDGGQGILGVCDTAATGYH	0.13	KARDGVQGILGVCDTAATGNH	0.11	KARDGVQGILGVCDTEATGYH	0.0
87	KARDGCTARAGSTVMGYYH	0.11	KARGGTGTSASTRCYFH	0.12	KARDGLKCTQRRSYSY	0.12	KARDGVQGILGVLDTAATGYH	0.11	KARDGVQGILGVCDTAATGYH	0.0
88	KARDGRVCAQLQGYYH	0.11	KARMGTATSCASTYYH	0.12	KARDGVHGILGVCARSYNYYH	0.12	KARDGVQGMLGVCDTAATGYH	0.11	KAGVYLSAVMHRCYYH	0.0
89	KAREGTDHG	0.11	KTHMGTATSCASTYYH	0.12	KARDGYMCVQLRDGYH	0.12	KAREGITACDSGGYYH	0.11	KARDARTPGSVGCSYRARGYH	0.0
90	KVRGGTGTSASTRCYYH	0.11	KARDGRVCAQLQGYYH	0.12	KAGVYLRAVMQRCYYH	0.12	KARDGVQGILGGWDTAATGYH	0.11	KARDARYGDINGGCGLKNH	0.0
91	KARDAGQKCVLAATVR	0.11	KTRLGTATSCTATYYH	0.12	KAQEHGYSDNVCGLQCVTDYY	0.12	KDRDGVQGILGVCDTAATGYH	0.11	KAGVYLSAVLQRCYYH	0.0
92	KARDGLRCTASRYYH	0.11	KPRGTRVCVQL	0.11	KAQEQGYMDGDCRVWGMCTGHYS	0.11	KAGVYLSAGMQRCYYH	0.11	KAGGYLSAVMQRCHYH	0.0
93	KARDGLRCTTSRYSH	0.11	RARDGGGHGVCASSSYYH	0.11	KARDARYGDINGVCGLKDH	0.11	KARDARTPGSVGCSYRDRGYH	0.11	KAGVYLIAVMQRCYYH	0.0
94	RARDGGGHGVCASSSYYH	0.11	RTRMGTATSCASTYYH	0.11	KARDARYGDINGVCGLKSH	0.11	KARDASTASGIAPVCVRGYYH	0.11	KAGVYLSSVMQRCYYH	0.0
95	KARDGGGHGVCASSSYYH	0.11	KAAKGSCSGNYY	0.11	KARDGLRCTTSRYYHDG	0.11	KARDGVQGILGGCDTAATGYQ	0.11	KAQDVRYGFRGCVWGDRCSDLYYY	0.0
96	KARDGGTARGSVCASAAYYH	0.11	KARDGLRFTTSRYYH	0.11	KARDGLRGTTSRYYH	0.11	KARDGVQGILGVCDTVATGYH	0.11	KARDASTASGIAPVCVRGYYH	0.0
97	KPRGTRVCVQL	0.11	KARGGTGTSASTRCYLH	0.11	KARGGTGTSASTRCYLH	0.11	KARDGVQGILGVCGTAATGYH	0.11	KARDGVQGILGVCDTAATGYP	0.0
98	KARGGEEVCVKLHTYH	0.10	KARDGGGNVCQHGDYH	0.11	KARMGTATSCASTYYH	0.11	NARDGVQGILGVCDTAATGYH	0.11	KARDARTPGSVGCSYRDRGCH	0.0
99	KARGGTGTRASTRCYYH	0.10	KARDGRLCVALQQDVH	0.11	KARDALVRGPGSCGNTQW	0.11	KAGVYLSAVMQRCYYQ	0.10	KAGGYLRAVMQRCYYH	0.0
100	KARDGRLCSTRNYYH	0.10	KARGGTGTSVSAGCYYH	0.11	KARDARYGDINGVCGLKIH	0.10	KAQSRVGRVCVGASCSGTYY	0.10	KAGVYLSAVMQRCDYH	0.0

Yeast display library

Ranking	Input Sequence	Input Abundance (%)	Round 1 Sequence	Round 1 Abundance (%)	Round 2 Sequence	Round 2 Abundance (%)	Round 3 Sequence	Round 3 Abundance (%)	Round 4 Sequence	Round 4 Abundance (%)
1	KAQSRVGRVCVGASCSGPYY	5.80	KAQSRVGRVCVGASCSGPYY	3.14	KAQEEWGPIQCHNSCSGAMNYY	15.83	KARDARYGDINGVCGLKNH	18.73	KARDARYGDINGVCGLKNH	31.94
2	KARGGTGTSASTRCYYH	4.11	KARGGTGTSASTRCYYH	2.52	KARDARYGDINGVCGLKNH	8.11	KAQEEWGPIQCHNSCSGAMNYY	13.28	KARDARTPGSVGCSYRDRGYH	10.23
3	KARDGLRCTTSRYYH	2.99	KARDGLRCTTSRYYH	2.31	KARDGVHGILGVCARSYNYYH	3.90	KARDGVHGILGVCARSYNYYH	7.60	KAQDVRYGFRGCVWGDRCSDLYYY	6.31
4	KARDGGGHGVCASSSYYH	2.61	KARDGGGHGVCASSSYYH	1.48	KAGVYLSAVMQRCYYH	2.72	KARDARTPGSVGCSYRDRGYH	4.52	KAQEEWGPIQCHNSCSGAMNYY	6.18
5	KARGGTGTSASTRCYYP	1.90	KAQKGSCRGNYY	1.38	KARDARTPGSVGCSYSDRGYH	1.44	KAGVYLSAVMQRCYYH	2.03	KARDGVHGILGVCARSYNYYH	4.96
6	KARHGGVGSCSSYYY	1.63	KAQEQGTAGVCVPRSCSGYYY	1.22	KARDARTPGSVGCSYRDRGYH	0.93	KAQDVRYGFRGCVWGDRCSDLYYY	1.30	KARDARYGDIKGVCGLKNH	0.76
7	KAQEQGTAGVCVPRSCSGYYY	1.61	KARGGTGTSASTRCYYP	1.00	KARDADPSVCANPQLWGYYH	0.90	KARDGVQGILRVCARATGGYYH	0.71	KAGVYLSAVMQRCYYH	0.71
8	KAQKGSCRGNYY	1.14	KARHGGVGSCSSYYY	0.83	KAQEEWGPIQCHNSCSGAMNYY	0.87	KARDARYGDSNGVCGLKNH	0.68	KARDARYGDSNGVCGLKNH	0.67
9	KARGGTGTSASTRCYYQ	1.12	KAQEEWGPIQCHNSCSGAMNYY	0.79	KAQEEWGPSQCHNSCSGAMNYY	0.84	KAQEEGGPIQCHNSCSGAMNYY	0.66	KAREARYGDINGVCGLKNH	0.67
10	KAQEPVYLSGIGCGADSCSGHYY	1.08	KARGGTGTSASTRCYYQ	0.79	KAQEEWGPIQWHNSCSGAMNYY	0.78	KARDARYGDINGVGGLKNH	0.60	KARDARDGDINGVCGLKNH	0.64
11	KAQEQGTAGVCVPRKCSGYYY	1.05	KAQEQGTAGVCVPRKCSGYYY	0.66	KAQEEWGPIQGHNSCSGAMNYY	0.74	KAQEEWGPIQCHNSCSGAMNYY	0.52	KARDARYGDINGVGGLKNH	0.59
12	KARGGTGTSASTRCYYR	0.94	KARGGTGTSASTRCYYR	0.56	KARDGVQGILGVCDTAATGYH	0.72	KARDARYGDIKGVCGLKNH	0.52	KARDARYGDINGVCGLKNH	0.56
13	KARMGTATSCSSTYYH	0.89	KARDARYGDINGVCGLKNH	0.53	KAQEEWGPIQCHNSCRGAMNYY	0.69	KARDADPRGCVQLQWGYYH	0.52	KARDGVQGILRVCARATGGYYH	0.55
14	KTRMGTATSCASTYYH	0.80	KAQEPVYLSGIGCGADSCSGHYY	0.49	KAQEEGGPIQCHNSCSGAMNYY	0.66	KAQEEWGPIQWHNSCSGAMNYY	0.50	KARDARYGDINGVCGLKNQ	0.52
15	KARGGTGTSVSAGCYYH	0.79	KAQEHGYSDNVCGLQCVPDYY	0.49	KAQEEWGPIQCHNSCSGARNYY	0.62	KAREARYGDINGVCGLKNH	0.50	KARDARYGDINGGCGLKNH	0.50
16	KARDGRLCVALQQDYH	0.78	KARGGTGTSVSAGCYYH	0.46	KAQEEWGPIQCQNSCSGAMNYY	0.56	KARDARYGDINGVCGLKNH	0.50	KAQDGRYGFRGCVWGDRCSDLYYY	0.45
17	KARDGGSLGSGRVCSTAGYYH	0.77	KARDGLKCTQQRSYYH	0.45	KAQEEWGPIQCHNSCSGAMNYY	0.49	KARDARYGDINGVCGLKNQ	0.49	KARDARYGEINGVCGLKNH	0.44
18	KAQEHGYSDNVCGLQCVPDYY	0.76	KARMGTATSCSSTYYH	0.42	KAQKGSCRGNYY	0.49	KAREGYKGDCRYTRYYH	0.49	KARDARYGDINGVCGLKNH	0.39
19	KAREGTDHGDDVCVTQFGAYYH	0.71	KAREGTDHGDDVCVTQFGAYYH	0.41	KAQSRVGRVCVGASCSGPYY	0.47	KAQEEWGPSQCHNSCSGAMNYY	0.47	KARDARYGDINGVCGVKNH	0.37
20	KARDGLKCTQQRSYYH	0.69	KARDGRLCVALQQDYH	0.41	KARDADPRGCVQLQWGYYH	0.47	KARDARYGDINGVCGLKNH	0.46	KARDARTPGSVGCSYRERGYH	0.35
21	KTRMGTATSCAATYYH	0.69	KARDGLRCTTSRYWH	0.36	KARGGTGTSASTRCYYH	0.45	KARDARDGDINGVCGLKNH	0.44	KAQDVRYGFRGWVWGDRCSDLYYY	0.34
22	KTREGTHTSCVSTNYH	0.49	KAGVYLSAVMQRCYYH	0.33	KAQEEWGPIQCHNSCSGAMNDY	0.42	KAQEEWGPIQCQNSCSGAMNYY	0.42	KARDARTPGRVGCSYRDRGYH	0.33
23	KARDGLRCTTSRYWH	0.46	KARDGVHGILGVCARSYNYYH	0.32	KAREGYKGDCRYTRYYH	0.37	KAQEEWGPIQCHNSCRGAMNYY	0.41	KARDARYGDINGVCGLKYH	0.32
24	KARGTRVCVQL	0.41	KARDGGSLGSGRVCSTAGYYH	0.30	KARDGVQGSIRVCERSTSGYYH	0.36	KAQEEWGPIQGHNSCSGAMNYY	0.41	KARDARYGDINGVCGWKNH	0.29
25	KARDGGTARGSVCASAAYYH	0.40	KTRMGTATSCASTYYH	0.30	KARDGVQGILRVCARATGGYYH	0.35	KAQEEWGPIQCHNSCSGARNYY	0.39	KAREARTPGSVGCSYRDRGYH	0.28
26	KARGGTGTSASTRCYYL	0.39	KTREGTHTSCVSTNYH	0.28	KARDARYGDINGVCGLKNQ	0.35	KARDGVQGILGVCDTAATGYH	0.39	KARDARYGDMNGVCGLKNH	0.28
27	KARMGTATSCAATYYH	0.36	KARGTRVCVQL	0.26	KARDARYGDINGVGGLKNH	0.31	KARDARYGDINGVCGVKNH	0.38	KARDARTPGSVGCSYRDRGYH	0.28
28	KAQARVGRVCVGASCSGPYY	0.34	KTRMGTATSCAATYYH	0.25	KARDGGGHGVCASSSYYH	0.31	KAQEEWGPIQCHNSCSGAMNYY	0.36	KARDARYGDINGVWGLKNH	0.26
29	KARDGRVCVRARLLH	0.33	KAQARVGRVCVGASCSGPYY	0.24	KARDARYGDIKGVCGLKNH	0.30	KARDARYGDINGVCGWKNH	0.36	KARGGTGTSASTRCYYH	0.25
30	KAREGTHTSCVSTNYH	0.32	KARDAEGVRDVCGTGYYH	0.23	KARDGLRCTTSRYYH	0.30	KARDGVHGILGVCARSYNYYH	0.35	KAQDVRYGCRGCVWGDRCSDLYYY	0.23
31	KARDAEGVRDVCGTGYYH	0.29	KARDGRVCVRLQGGLPH	0.23	KARDGVHGSLGVCARSYNYYH	0.28	KARDGVHGILGGCARSYNYYH	0.35	KAQEEWGPIQCHNSCRGAMNYY	0.23
32	KARDGRVCVRLQGGLPH	0.27	KAQSRVGRGCVGASCSGPYY	0.23	KAREARYGDINGVCGLKNH	0.26	KARGTRVCVQL	0.35	KARDARTPGSVGGSYRDRGYH	0.23
33	KARDGKLCVALQQDYH	0.27	KAQSRVGRVGVGASCSGPYY	0.23	KARDARYGDSNGVCGLKNH	0.26	KARDARYGDINGGCGLKNH	0.33	KAQDVRYGFRGCVGGDRCSDLYYY	0.22
34	KARDGLRCTTSRYYY	0.26	KAQSRVGRVCVGASCRGPYY	0.22	KAQEEWGPIQCHNSGSGAMNYY	0.25	KAQEEWGPIQCHNSGSGAMNYY	0.28	KAKDVRYGFRGCVWGDRCSDLYYY	0.22
35	KAQKGSCRGIYY	0.26	KARDARTPGSVGCSYSDRGYH	0.21	KARDGVHGILGGCARSYNYYH	0.25	KARDARYGEINGVCGLKNH	0.28	KAQDVRYGFRGCVWGDRCSDLYYY	0.22
36	KAQSRVGRVGVGASCSGPYY	0.25	KARDGRVCVRARLLH	0.21	KARGGFVRCVLTATVGDYH	0.25	KARDGVHGILGVCARSYNDYH	0.27	KAQEEGGPIQCHNSCSGAMNYY	0.21
37	RARMGTATSCASTYYH	0.25	KARGGTGTSASTRCYYH	0.20	KAQEEWGPIQCHKSCSGAMNYY	0.23	KARDARYGDINGVCGLKYH	0.27	KARDGVQGILGVCDTAATGYH	0.21
38	KARMGTATSCAASYYH	0.25	KARMGTATSCAATYYH	0.19	KARDGVHGILGVCARSYNYYH	0.23	KARDARYGDI	0.27	KARDARAPGSVGCSYRDRGYH	0.21
39	KARVGGDCRVSSTYGYYH	0.24	KAQKGSCRGIYY	0.18	KARDARYGEINGVCGLKNH	0.23	KAQEEWGPIQCHNSCSGAMNDY	0.25	KAQEEWGPIQWHNSCSGAMNYY	0.21
40	KGRGGTGTSASTRCYYH	0.22	KARDGKLCVALQQDYH	0.18	KARGGTGTSASTRCYYP	0.23	KARDGVHGIRGVCARSYNYYH	0.25	KARDARYGDINGVCGLKDH	0.21
41	KAQEQGTAGVCVPRSCSGHYY	0.21	KARDGLRCTTARYWH	0.17	KAQEEWGPIQFHNSCSGAMNYY	0.19	KAQDGRYGFRGCVWGDRCSDLYYY	0.22	KAQDVRYGFRGGVWGDRCSDLYYY	0.20
42	KARDGGGHGVCASSSYYH	0.21	KAQSRVGRVCVGASCSGPYY	0.16	KARDGVHGILGVWARSYNYYH	0.19	KARDGVHGSLGVCARSYNYYH	0.22	KARDGVHGILGGCARSYNYYH	0.20
43	KARGGTGTSASTRCYYH	0.21	KARDGGTARGSVCASAAYYH	0.16	KARDGVHGIRGVCARSYNYYH	0.19	KARDARTPGSVGCSYRERGYH	0.20	KAQEEWGPSQCHNSCSGAMNYY	0.20
44	KAQSRVGRGCVGASCSGPYY	0.20	KAQEHRTCPRGGSCSGPYY	0.16	KARDARYGDINGGCGLKNH	0.19	KARDGVHGILGVCARSYNYYQ	0.20	KARDGGGHGVCASSSYYH	0.20
45	KAQSRVGRVCVGASCSGPYY	0.20	KAREGTHTSCVSTNYH	0.16	KAQEEWGPIQCHNSCSGAINYY	0.18	KARDARTPGSVGCSDRDRGYH	0.20	KAREGYKGDCRYTRYYH	0.20
46	KARGGTGTRASTRCYYH	0.20	KARDADGEGVCAERYYH	0.15	KAQEEWGQIQCHNSCSGAMNYY	0.18	KARDARYGDMNGVCGLKNH	0.20	KAQEEWGPIQCHNSCSGAMNYY	0.19
47	KTRGGTGTSASTRCYYH	0.20	KARDGLRCTTSRYYY	0.15	KAQEEWVPIQCHNSCSGAMNYY	0.18	KARDARYGDINGVWGLKNH	0.20	KARDARTPGSVGCSDRDRGYH	0.19
48	KAQSRVGRVCVGASCRGPYY	0.19	KAQSRVGRVCVGASGSGPYY	0.14	KARDARYGDINGVCGLKNH	0.18	KARGGTGTSASTRCYYH	0.20	KAQEEWGPIQGHNSCSGAMNYY	0.19
49	KARDGGYSVQVCSSAGYYH	0.19	KARGGTGTSASTRCYYL	0.14	KARDARDGDINGVCGLKNH	0.18	KARDGGHGILGVCARSYNYYH	0.19	KARDGVHGILGVCARSYNYYH	0.19
50	KARYDGGTGCAATVSHYH	0.19	KARDGRSCTGLRGHYH	0.14	KARGGTGTSVSAGCYYH	0.18	KARDGVHGILGVCARSDNYYH	0.19	KARDARTPGSGGCSYRDRGYH	0.18
51	KAQSRVGRVCVGASCSGPYY	0.18	KAQEGGGVCGRSCSGPYY	0.13	KAQEEWGPIHCHNSCSGAMNYY	0.17	KAQEEWGPIQCHNSCSGAINYY	0.17	KARDARTPGSVGCSYRDRGYQ	0.17
52	KARDGCTARAGSTVMGYYH	0.18	RARDGGGHGVCASSSYYH	0.13	KAQEDWGPIQCHNSCSGAMNYY	0.17	KARDGVHGILGVWARSYNYYH	0.17	KARDARYGDI	0.17
53	KAREGTVVDVCVEGYYH	0.17	KARDGLRCTTARYYH	0.13	KARDGVHGILGVCARSYNDYH	0.17	KARDARYGDINGVCGLKDH	0.17	KARDGVHGIRGVCARSYNYYH	0.17
54	KARGGTGTSASTRCYYN	0.17	KARDGLRCTTSRYYQ	0.13	KARDGGHGILGVCARSYNYYH	0.17	KARDGVHGMLGVCARSYNYYH	0.16	KAQDVRYGFRGCGWGDRCSDLYYY	0.16
55	KAQEGGGVCGRSCSGPYY	0.17	KARGGTGTRASTRCYYH	0.13	KARDARYGDINGVCGLKNH	0.17	KARDARTPGSVGGSYRDRGYH	0.16	KAQEEWGPIQCQNSCSGAMNYY	0.16
56	KAQGRTGLCVGSCSGGYY	0.17	KARDGGGNVCQHGDYH	0.13	KAQEEWGPIQCHNSCSCAMNYY	0.16	KARDARTPGSVGCSYRDRGYH	0.14	KARGTRVCVQL	0.16
57	KAQEHRTCPRGGSCSGPYY	0.16	GAQKGSCRGNYY	0.13	KAQEQGTAGVCVPRSCSGYYY	0.16	KARDARTPGRVGCSYRDRGYH	0.14	KARDARPPGSVGCSYRDRGYH	0.16
58	KAQEQRRYDGDTCVLGLCILNYY	0.14	KAQSRVGRVCVGASCSGPYY	0.12	KARDGVHGILGVCARSYNYYH	0.16	KAREARTPGSVGCSYRDRGYH	0.14	KAQSRVGRVCVGASCSGPYY	0.16
59	KARDAGTALDVCATVGPSYYH	0.14	KAQSRVGRVWVGASCSGPYY	0.12	KARDARYGDINGVCGVKNH	0.16	KARDARTPGSVGCSYRDRGYQ	0.14	KARDADPRGCVQLQWGYYH	0.16
60	KARGGTGTSASTRCYYH	0.14	KARDGGGHGVCASSSYYH	0.12	KARDGRLCVALQQDYH	0.16	KARDADPSVCANPQLWGYYH	0.14	KARDARYGDINGVCGFKNH	0.15
61	KAQSRVGRVCVGASGSGPYY	0.14	KARVGGDCRVSSTYGYYH	0.12	KAQEEWGPIQCHNSCSGAMNYY	0.14	KARHGGVGSCSSYYY	0.14	KAQEEWGPIQCHNSCSGAMNYY	0.15
62	KAQSRGGRVCVGASCSGPYY	0.14	RARMGTATSCASTYYH	0.12	KSQEEWGPIQCHNSCSGAMNYY	0.14	KAQKGSCRGNYY	0.14	KARDARYGDINGVCGLKHH	0.15
63	KARMGTATSCATTYYH	0.14	KARDGTRHDRVCRVPGDRAGHYH	0.11	KARDARYGDINGVCGLKYH	0.14	KAQDVRYGFRGCGWGDRCSDLYYY	0.13	KARDARTPGSVGCSYRDRGYH	0.13
64	RAREGTHTSCVSTKYH	0.14	KARDADPSVCANPQLWGYYH	0.11	KARDARYGDINGVWGLKNH	0.14	KAQEEWGPIQFHNSCSGAMNYY	0.13	KAQEEWGPIQCHNSCSGAMNDY	0.13
65	KTRVGGDCRVSSTYGYYH	0.13	KARDGGGHGVCASSSYYH	0.11	KARDARYGDI	0.14	KARDGVQGSIRVCERSTSGYYH	0.13	KARDGVHGILGVCARSYNYYQ	0.13
66	KGQEQGTAGVCVPRSCSGYYY	0.13	KGRGGTGTSASTRCYYH	0.11	KARGTRVCVQL	0.14	KAQEEWGPIQCHNSCSGAMNYY	0.13	KAQDVRYGFRGCVWGDRCSDLYYY	0.12
67	KARGGTGTSASTRCYYP	0.13	KTRGGTGTSASTRCYYH	0.11	KARHGGVGSCSSYYY	0.13	KARDGVHGILGVCARSYNYYH	0.13	KAQDVRYGVRGCVWGDRCSDLYYY	0.11
68	KARMGTATSCATSYYR	0.13	KARDGLRCTTSRYDH	0.11	KAQEEWGPIRCHNSCSGAMNYY	0.13	KAREGVHGILGVCARSYNYYH	0.13	KAQEEWGPIQCHNSCSGARNYY	0.11
69	KAQEQRRYDGDTCVLGLCIVNYY	0.12	KAQKGSGRGNYY	0.11	KAQEELGPIQCHNSCSGAMNYY	0.13	KARDARYGDINGVCGLKHH	0.13	KARDGVHGILGVGARSYNYYH	0.11
70	KARDGRVQRGSYVCVTSTAH	0.12	KAQKGSCRGNYY	0.11	KARDGGTARGSVCASAAYYH	0.13	KARDARYGDINGVCGLNNH	0.13	KAQAVRYGFRGCVWGDRCSDLYYY	0.10
71	KARGGTGTSASTTCYYH	0.12	KAQEEWGPIQCHNSCSGAMNYY	0.10	KARGGTGTSASTRCYYQ	0.13	KSRDARYGDINGVCGLKNH	0.13	KAQDVRYGLRGCVWGDRCSDLYYY	0.10
72	KTRMGTATSCAASYYH	0.12	KAQSRVGRVCGGASCSGPYY	0.10	KAQDVRYGFRGCVWGDRCSDLYYY	0.12	KAQEEWGPIQCHNSCSGAMNYD	0.11	KARDARTPGSVGCSYRDRGDH	0.10
73	KARMGTATSCASTYYH	0.12	KAQGRTGLCVGSCSGGYY	0.10	KAQEEWGPIQCHNICSGAMNYY	0.12	KAQEEWVPIQCHNSCSGAMNYY	0.11	KAQDVRYGFRGCVWGDRGSDLYYY	0.10
74	KAQEHRDCGCVVLQWPHYY	0.12	KAQEHRGRMCVASCSGNY	0.10	KARDGVHGMLGVCARSYNYYH	0.12	KARDARTPGSGGCSYRDRGYH	0.11	KARDGVHGSLGVCARSYNYYH	0.10
75	KARDADGEGVCAERYYH	0.12	KARGGTGTSASTRCYDH	0.10	KAQEECGPIQCHNSCSGAMNYY	0.11	KARDGVHGILGVCARSYNYDH	0.11	KARDGVHGILGVWARSYNYYH	0.10
76	KARDAGQKCVLAATVR	0.12	RAREGTHTSCVSTKYH	0.10	KAQEEWGPIQCQNSCSGAMNDY	0.11	KARDARTPGSVGCSYSDRGYH	0.11	KARDAGYGDINGVCGLKNH	0.10
77	KARDGGGNVCQHGDYH	0.12	KARDGRLCVSVQGDYH	0.10	KARDGVHGILGVCARSDNYYH	0.11	KARDARYGDINGVCGLKNH	0.11	KARDGRLCVALQQDYH	0.10
78	KARDGLRCTTSRYDH	0.12	KARDGLRCTTSRYSH	0.10	KAGVYLRAVMQRCYYH	0.11	KARDARYGDINGVCGFKNH	0.11	KAQDVRDGFRGCVWGDRCSDLYYY	0.09
79	KAQSRVGRVCVGASCSGPDY	0.11	KARDGTRHDRVCRVPSDRAGHYH	0.09	KAGVYLSAVMQRCYDH	0.11	KDRDARYGDINGVCGLKNH	0.11	KAREGVHGILGVCARSYNYYH	0.09
80	KAQERGRPCVGSCSAYYY	0.11	KAQEQGTAGVCVPRSCSGHYY	0.09	KAQEPVYLSGIGCGADSCSGHYY	0.10	KARDARYVDINGVCGLKNH	0.11	KSRDARYGDINGVCGLKNH	0.09
81	KTHMGTATSCASTYYH	0.11	KAQSRGGRVCVGASCSGPYY	0.09	KARDGVHGILGVGARSYNYYH	0.10	KAQDVRYGFRGWVWGDRCSDLYYY	0.09	KAQEPVYLSGIGCGADSCSGHYY	0.09
82	RARMGTATSCATTYYH	0.11	KARDAGTRVCPSYGKGYYH	0.09	KARDGVHGILGVCARSYNYYQ	0.10	KAQEEWGPVQCHNSCSGAMNYY	0.09	KARDGVHGILGVCARSYNYYH	0.09
83	KARDGLRCTTARYYH	0.11	KARDAVHGDCASGSVYYH	0.09	KARDARTPGSVGCSYRERGYH	0.10	KAQEECGPIQCHNSCSGAMNYY	0.09	KARDGLRCTTSRYYH	0.09
84	KARDGGSLGSGRVCSTAGYYH	0.11	KARMGTATSCAASYYH	0.09	KARDARYGDINGVCGLKHH	0.10	KARDARAPGSVGCSYRDRGYH	0.09	KARDGVHGMLGVCARSYNYYH	0.08
85	KARGLRTARGLSCSSHYYH	0.11	KAAKGSCSGNYY	0.09	KARDARYGDINGVCGWKNH	0.10	KARDARTPGSVGCSYRDRGYH	0.09	KARDARYGDINGVCGLNNH	0.08
86	KARDGLKCTHERSYYH	0.11	KAQKGSCKGNYY	0.09	KARDARYGDMNGVCGLKNH	0.10	KARDGVPGILGVCARSYNYYH	0.09	KAREGYSAGLGCVRGGYH	0.08
87	KARDAGTRVCPSYGKGYYH	0.10	KAQEEWGPIQCHNSCSGAMNYY	0.08	KAQEEGGPSQCHNSCSGAMNYY	0.08	KARDGVHGILGVCARSYNYSH	0.09	KAQEQCGRRCSGDYY	0.08
88	KARDGGGHGGCASSSYYH	0.10	KARDARTPGSVGCSYRDRGYH	0.08	KAQEEGGPIQCHNSCSGAMNYY	0.08	KARDGVHGILGVGARSYNYYH	0.09	KARDGVHGILGVCARSYNDYH	0.08
89	KARDGRLCVSVQGDYH	0.10	KARDGCTARAGSTVMGYYH	0.08	KAQEEWGPIQCHNSCSVAMNYY	0.08	KAQSRVGRVCVGASCSGPYY	0.09	KARDARYGDINGVCGLKTH	0.08
90	KVRHGGVGSCSSYYY	0.10	KARDGGYSVQVCSSAGYYH	0.08	KAQEEWGPIQCHNSCSGAMNYH	0.08	KARDGGGHGVCASSSYYH	0.09	KAQDVRYGFRGCVWGDRCSDLYDY	0.07
91	KAQEQGYMDGDCRVWGMCTGHYS	0.10	KARDGGGHGVCAASSYYH	0.08	KAQEEWGPIQWHNSCSGAMNYY	0.08	KAKEEWGPIQCHNSCSGAMNYY	0.08	KARDARYGDINGVCGLKNH	0.07
92	KAQEQGTAGVCVPRRCSGYYY	0.10	KARGGTGTSASTRCYYP	0.08	KAQEEWGPIQCHNSCSGAMNYD	0.08	KAQEEWGPIQCHNSCSGAMTYY	0.08	KAQDVRYGFRGCVWGDRCRDLYYY	0.06
93	KAQSRVGRVCGGASCSGPYY	0.10	KARGGTGTSASTRCYYP	0.08	KAREEWGPIQCHNSCSGAMNYY	0.08	KAQEEWGPIQCHNSCSCAMNYY	0.08	KARDGGHGILGVCARSYNYYH	0.06
94	KAQEHRGRMCVASCSGNY	0.10	KARGGTGTSASTRWYYH	0.08	KARDGVPGILGVCARSYNYYH	0.08	KAQEEWGPIHCHNSCSGAMNYY	0.08	KARDARYVDINGVCGLKNH	0.06
95	KARDDDLCVRLHVGGYYH	0.10	KARDGLKCTRERSYYH	0.08	KAREGVHGILGVCARSYNYYH	0.08	KARDARSPGSVGCSYRDRGYH	0.08	KARDDRYGDINGVCGLKNH	0.06
96	KARDAVHGDCASGSVYYH	0.10	KAQEPGPSGIAHVCGRCSGGYYY	0.08	KAREARTPGSVGCSYSDRGYH	0.08	KARYGVHGILGVCARSYNYYH	0.08	KAQDARYGDINGVCGLKNH	0.06
97	RARDGGGHGVCASSSYYH	0.10	KAQEEGGPIQCHNSCSGAMNYY	0.08	KARDARYGDINGVCGFKNH	0.08	KARDARYGYINGVCGLKNH	0.08	KARMGTATSCTSYYH	0.06
98	KAQEQAVCGRRSCSGDYY	0.10	KGQEQGTAGVCVPRSCSGYYY	0.08	KARDARYGDINGVCGLKTH	0.08	KARDARYGDINVVCGLKNH	0.08	KAQDVRYGFRGCVWGDRCSDLDYY	0.05
99	KARDGLKCTRERSYYH	0.10	KAQEQGTAGVCVPRSCSGYYY	0.08	KAGVYLSAVMQRCYYH	0.08	KARDARYGDINGVCGLKSH	0.08	KAQDVRYGFRGCVWGDRCSELYYY	0.05
100	KARGGTGTSASTRCYLH	0.09	KARDGGSRGSGRVCSTAGYYH	0.08	KAGVYLSAGMQRCYYH	0.08	KARGGTGTSASTRCYYQ	0.08	KARDGVQGSIRVCERSTSGYYH	0.05

Supplementary Table S2. List of major CDR3 sequences before and after biopanning.

Y, yeast; P, phage; R0, input library; R4, concentrated library by 4-round biopanning; last number, ranking of abundance.

ND : not detected.

				Deep sequencing									
				Abundance (fraction of total sequences, %)									
				Phage display					Yeast display				
CDR3	Sequence	Length	Cys	Input	Round 1	Round 2	Round 3	Round 4	Input	Round 1	Round 2	Round 3	Round 4
P-R0-01 / Y-R0-03	KARDGLRCTTSRYYH	15	1	6.29	5.73	4.41	0.33	0.09	2.99	2.31	0.30	0.05	0.09
P-R0-02 / Y-R0-02	KARGGTGTSASTRCYYH	17	1	6.29	6.72	3.43	0.16	0.04	4.11	2.52	0.45	0.20	0.25
P-R0-03 / Y-R0-06	KARHGGVGSCSSYYY	15	1	2.84	2.48	1.59	0.06	0.02	1.63	0.83	0.13	0.14	0.03
P-R0-04 / Y-R0-08	KAQKGSCRGNYY	12	1	2.72	3.16	1.97	0.04	0.01	1.14	1.38	0.49	0.14	0.00
P-R0-05 / Y-R0-09	KARGGTGTSASTRCYYQ	17	1	2.30	2.22	1.56	0.11	0.02	1.12	0.79	0.13	0.08	0.02
P-R0-06 / Y-R0-04	KARDGGGHGVCASSSYYH	18	1	1.97	1.85	0.78	0.08	0.01	2.61	1.48	0.31	0.09	0.20
P-R0-07	KARDGLKCTQQRSYYH	16	1	1.80	1.85	0.81	0.02	0.00	0.69	0.45	0.05	ND	ND
P-R0-08	KARDGRLCVALQQDYH	16	1	1.39	1.32	0.60	0.03	0.00	0.78	0.41	0.16	0.02	0.10
P-R0-09	KARGGTGTSASTRCYYHE	18	1	1.25	1.32	0.88	0.02	ND	0.21	0.20	0.05	0.02	0.00
P-R0-10	KTRMGTATSCASTYYH	16	1	1.14	0.97	0.84	0.05	0.02	0.80	0.30	0.05	ND	ND
Y-R0-01	KAQSRVGRVCVGASCSGPYY	20	2	0.18	0.18	0.16	0.02	0.01	5.80	3.14	0.47	0.09	0.16
Y-R0-05	KARGGTGTSASTRCYYP	17	1	0.93	0.91	0.48	0.07	0.02	1.90	1.00	0.23	0.02	0.04
Y-R0-07	KAQEQGTAGVCVPRSCSGYYY	21	2	0.84	0.79	0.28	0.03	0.01	1.61	1.22	0.16	0.02	0.01
Y-R0-10	KAQEPVYLSGIGCGADSCSGHYY	23	2	0.13	0.20	0.04	0.02	0.01	1.08	0.49	0.10	0.02	0.09
P-R4-01	KARDGVQGILGVCDTAATGYH	21	1	0.08	0.18	15.26	63.65	31.65	ND	0.03	0.72	0.39	0.21
P-R4-02 / Y-R4-07	KAGVYLSAVMQRCYYH	16	1	0.03	0.13	7.25	6.27	26.92	0.00	0.33	2.72	2.03	0.71
P-R4-03 / Y-R4-02	KARDARTPGSVGCSYRDRGYH	21	1	0.01	0.00	0.22	2.04	15.21	ND	0.08	0.93	4.52	10.23
P-R4-04	RAQDVRYGFRGCVWGDRCSDLYYY	24	2	ND	ND	0.13	0.44	2.70	ND	ND	0.53	ND	ND
P-R4-05 / Y-R4-01	KARDARYGDINGVCGLKNH	19	1	0.01	0.20	2.91	2.12	2.49	ND	0.53	8.11	18.73	31.94
P-R4-06	KARDARYGDIN	11	0	0.01	0.02	1.62	0.73	0.99	ND	0.01	0.14	0.27	0.17
P-R4-07	KAGGYLSAVMQRCYYH	16	1	ND	0.01	0.18	0.20	0.88	ND	0.01	0.02	0.02	0.01
P-R4-08	KAGVYLSAVMQRCHYH	16	1	0.00	0.00	0.15	0.13	0.72	ND	ND	0.53	ND	ND
P-R4-09	KARDGVQGILGVWDTAATGYH	21	0	ND	0.01	0.32	1.29	0.71	ND	0.01	0.53	ND	0.00
P-R4-10	KARDGVQGILGGCDTAATGYH	21	1	ND	ND	0.21	1.02	0.57	ND	ND	0.01	ND	0.00
Y-R4-03	KAQDVRYGFRGCVWGDRCSDLYYY	24	2	ND	ND	0.04	0.03	0.03	ND	ND	0.12	1.30	6.31
Y-R4-04	KAQEEWGPIQCHNSCSGAMNYY	22	2	0.01	0.04	0.01	ND	ND	0.05	0.79	15.83	13.28	6.18
Y-R4-05	KARDGVHGILGVCARSYNYYH	21	1	0.00	0.01	0.11	0.02	0.01	0.01	0.32	3.90	7.60	4.96
Y-R4-06	KARDARYGDIKGVCGLKNH	19	1	ND	ND	0.01	0.00	0.00	ND	0.04	0.30	0.52	0.76
Y-R4-08	KARDARYGDSNGVCGLKNH	19	1	ND	ND	0.01	0.01	0.01	ND	0.02	0.26	0.68	0.67
Y-R4-09	KAREARYGDINGVCGLKNH	19	1	ND	ND	ND	0.00	0.00	ND	0.01	0.26	0.50	0.67
Y-R4-10	KARDARDGDINGVCGLKNH	19	1	ND	ND	0.01	0.00	0.00	ND	ND	0.18	0.44	0.64
